# A Comparative Study Based on HS-SPME-GC-MS of Volatile Compounds in Large Yellow Croaker (*Pseudosciaena crocea*) During Varied Cold Storage Conditions

**DOI:** 10.3390/foods14122063

**Published:** 2025-06-11

**Authors:** Wenyuchu Chen, Fang Tian, Ailing Cao, Weiliang Guan, Tianyu Liu, Ying Liu, Luyun Cai

**Affiliations:** 1 Ningbo Global Innovation Center, Zhejiang University, Ningbo 315100, China; chenwenyuchu@zjou.edu.cn (W.C.); tianfang@zjou.edu.cn (F.T.); 10913029@zju.edu.cn (T.L.); 2 Key Laboratory of Health Risk Factors for Seafood of Zhejiang Province, School of Food and Pharmacy, Zhejiang Ocean University, Zhoushan 316022, China; 3 Hangzhou Customs District, Hangzhou 310008, China; 4 School of Light Industry and Food Engineering, Guangxi University, Nanning 530004, China; 5 College of Biosystem Engineering and Food Science, Zhejiang University, Hangzhou 310058, China

**Keywords:** slurry ice, *Pseudosciaena crocea*, HS-SPME-GC-MS, volatile compound profiling

## Abstract

Various volatile compounds are responsible for the odor changes in fish during storage. In this study, a coupled headspace solid-phase microextraction (HS-SPME) and gas chromatography–mass spectrometry (GC-MS) analytical approach was applied to characterize the volatile compounds in large yellow croaker (*Pseudosciaena crocea*) during storage under three treatments: cold storage (CS), slurry ice (SI), and crushed ice (CI). A total of 24 volatile substances were identified, including aldehydes, ketones, and alcohols. Multivariate statistical analyses (PCA, PLS-DA, VIP, and cluster heatmap) revealed significant differences in volatile compounds between the treatment groups during storage, and 10 key volatiles along with 5 potential biomarker compounds were identified. The underlying mechanisms of volatile changes were further investigated by analyzing three key pathways: thermal reactions, lipid oxidation, and amino acid degradation. Notably, SI treatment better avoid volatile compound variation in large yellow croaker.

## 1. Introduction

Large yellow croaker (*Pseudosciaena crocea*) is one of the most important economic fish products in the Asia–Pacific region [1,2]. In 2021, the production of large yellow croaker from mariculture was 254,224 tons, 13.79% of which was cultured in China [3]. Fish is recognized as a highly nutritious food source, rich in proteins, unsaturated fatty acids, and various essential nutrients. However, its high moisture and nutrient content make it highly perishable and prone to the development of off-odors during storage and handling [4]. Odor is a major factor in consumers’ perception of the freshness of fish [5].

Post-harvest large yellow croakers are usually exposed to different degrees of stress directly after being exposed to the air or at low temperatures, and the resulting stresses have significant impacts on flesh quality [6]. Commonly, fishermen slaughter the fish directly after capture with a blow to the head and subsequently add CI or chemical additives to preserve the freshness of the fish [7,8]. However, these methods will not only increase the labor consumption and economic costs of merchants but also increase the pain suffered by the fish. Therefore, there is a demand for a convenient and efficient method of preserving freshness and protecting the welfare of fish.

Slurry ice (SI), a fluid composed of liquid and small spherical ice crystals, can be made directly from seawater [9]. The large number of ice crystals in SI shows an extensive surface area for heat transfer, while the large enthalpy change and small temperature fluctuations that occur during melting result in a fast cooling rate [10]. Immersing post-harvest gilthead seabream directly in seawater SI required a shorter freezing time than placing them in freshwater crushed ice, which delayed fish spoilage during refrigeration [11]. Additionally, the unique structure of the SI isolates oxygen in air from aquatic products, slowing down the oxidation and inhibiting the microorganism’s propagation, which shows that it is effective in the preservation of freshness of fish and crustaceans [12,13]. Previous studies have investigated the effect of SI as a storage medium for shrimp (*Penaeus vannamei*) and found that SI not only inhibited microbial growth and slowed the growth of TVB-N but also protected lipids and proteins from oxidative damage [8,14]. However, current studies on SI preservation have focused on muscle protein structure, lipid oxidation, and microbial colonization, with little exploration of the effect on odor. This resulted in improved water retention and better textural properties of the shrimp. However, the changes in volatile compounds during storage remain inadequately explained and require further investigation.

In recent years, headspace solid-phase microextraction (HS-SPME) coupled to gas chromatography–mass spectrometry (GC-MS) has attracted considerable attention for its simplicity, sensitivity, and effectiveness in the analysis of complex odor compounds in food systems [15,16]. It is important to highlight that it does not measure complex food odor components; it measures volatile compounds, some of which can be related to certain aromas. The technique allows volatiles that are different between the white and yellow croaker, which are very similar in appearance, to be detected and quantified, as well as the identification of different characteristic volatile compounds in raw and cooked large yellow croaker [17,18]. However, most of the current research has been directed at identifying key volatile compounds in aquatic products, and only a little information is known about the relationship between storage conditions and volatile compounds. The application of volatile organic compound analysis in the food field has enabled the investigation of chemical changes in food products and their impact on the quality characteristics of the final product [19]. Various biochemical processes, including oxidation, microbial activity, and enzymatic reactions, contribute to the formation of diverse volatile compounds in fish meat, ultimately leading to changes in odor. Previous studies have investigated the volatile compounds in large yellow croaker at various storage stages using headspace–gas chromatography ion mobility spectrometry (HS-GC-IMS) combined with principal component analysis (PCA), demonstrating the effectiveness of this approach in odor profiling [20]. Building on this, other researchers have also employed GC-IMS as a rapid and sensitive analytical tool to further investigate the changes in volatile compounds of large yellow croaker subjected to different post-harvest treatments [21]. However, the relationship between storage conditions and the formation of these volatile compounds of CI, SI, and low temperature as different storage environments is not defined [22].

This study aims to identify the characteristic volatile compounds and investigate their formation mechanisms in large yellow croaker under different storage conditions by HS-SPME-GC-MS and combined with chemometric analysis. It will provide a theoretical basis for the research of volatile generation mechanisms and subsequent market regulation in the aquatic products industry.

## 2. Material and Methods

### 2.1. Experimental Design and Sampling

Large yellow croaker with an average weight of 500 ± 50 g (for each fish) was purchased at a local farm (Zhejiang, China). They were moved into normally oxygenated seawater tanks immediately and transported to the laboratory alive. The fish were held temporarily at approximately 25 °C for 24 h to reduce the stress associated with the transport process. The methodology is slightly modified from [23]; the fish were randomly divided into three groups with three different storage methods for 12 days. Cold storage group (CS): Five fish were placed directly into pre-cooled (2 °C) expanded polypropylene (EPP) (430 × 210 × 270 mm^3^) boxes (21 L) stored in an artificial climate chamber at 2 °C. Slurry ice group (SI): Five fish were evenly immersed into seawater slurry ice in the EPP box and stored in an artificial climate chamber at 2 °C. Crushed ice group (CI): Five fish and freshwater crushed ice were evenly distributed in the EPP box and stored in an artificial climate chamber at 2 °C. Large yellow croaker fillets from all groups were collected at 0 d, 6 d, and 12 d, respectively, and placed in polyethylene (PE) plastic bags. The 0-day (0 d) group designates fish fillet samples subjected to 2 h of storage, serving as the initial time point for comparative analysis. Three samples were randomly selected from each group at each time for analysis to reduce individual differences. The CS treatments were set to C0, C6, and C12; the SI treatments were set to L0, L6, and L12; and the CI treatments were set to P0, P6, and P12.

### 2.2. Extraction of Volatile Compounds

The extraction of large yellow croaker volatile compounds was carried out according to Zhang et al., (2019) [17] with some modifications. The PAL System (CTC Analytical Instruments Co., Ltd., Basel, Switzerland) was used for solid-phase microextraction auto sampling. The sample was finely chopped and accurately weighed 5.0 g in a 20 mL headspace glass vial with a polytetrafluoroethylene septum. Subsequently, 80 µL of 6 mg/L 2,4,6-trimethylpyridine was added as an internal standard and sealed. After equilibration in a shaker at 25 °C for 20 min, extraction with activated 50/30 µm DVB/CAR/PDMS SPME fibers was carried out at 60 °C for 50 min. Finally, desorption was carried out at 250 °C for 5 min.

### 2.3. Volatile Compound Profiling

Samples were analyzed using an Agilent 8890 gas chromatography system and an Agilent single quadrupole 5977 gas–mass spectrometer (Agilent Technologies, Santa Clara, CA, USA). Separation of compounds were carried out on a non-polar HP-5MS capillary column (30 m, 0.250 mm ID, and 0.25 µm film thickness). Helium was used as the carrier gas and run in splitless mode with the flow rate set to 1.0 mL/min. The initial temperature of the program was 30 °C, maintained for 3 min, then the temperature was raised to 120 °C at a rate of 8 °C/min, then to 250 °C at a rate of 15 °C/min, and finally maintained at that temperature for 3 min. The ion source temperature was 230 °C, the quadrupole temperature 150 °C, the transfer line temperature 250 °C, and the quality scan range was 45–550. Identification of volatile compounds was performed by comparing the acquired mass spectra with those in the NIST 17 mass spectral library. Only compounds with a similarity index (match quality) of ≥85% were considered tentatively identified. For additional confirmation, linear retention indices (LRIs) were calculated using a standard series of n-alkanes (C7–C30) analyzed under the same chromatographic conditions, and compared with reference values reported in the literature. For peak detection, the initial threshold was maintained at 12, with the initial peak width and initial area reject set to 0.2 and 1, respectively.

### 2.4. Statistical Analysis

SPSS version 25.0 software for Windows (SPSS Inc., Chicago, IL, USA) was used for correlation tests and ANOVA for analysis of variance, with significance defined as *p* < 0.05. The raw GC-MS data were normalized by applying logarithmic transformation and Pareto. Multidimensional statistical analysis was performed using the online platform tool Metaboanalyst 6.0 (www.metaboanalyst.ca) (accessed on 1 December 2023). Relative peak areas were differentiated by principal component analysis (PCA) and orthogonal partial least squares discriminant analysis (OPLS-DA). Volatile compounds with Variance Importance Prediction (VIP) values exceeding 1 are important indicators of odor profile variations. Loading plots, box plots, and heatmaps were drawn using Excel and Origin 8.5.

## 3. Results and Discussion

### 3.1. Volatile Compound Analysis: Component Identification

As shown in Table 1, the identified compounds are listed with their respective molecular formulas, abbreviations, CAS numbers, and retention times. These results identified a total of 24 volatile compounds, which were categorized into nine structural groups: (2) nitrogen compounds, (2) aldehydes, (8) hydrocarbons, ketones, ethers, alcohols, phenols, esters, and (7) others. These findings indicate that distinct volatile compounds can be effectively separated and identified from large yellow croaker during storage, and the corresponding mass spectra are provided in the Supporting Material.

### 3.2. Multivariate Data Analysis

**Figure 1:**
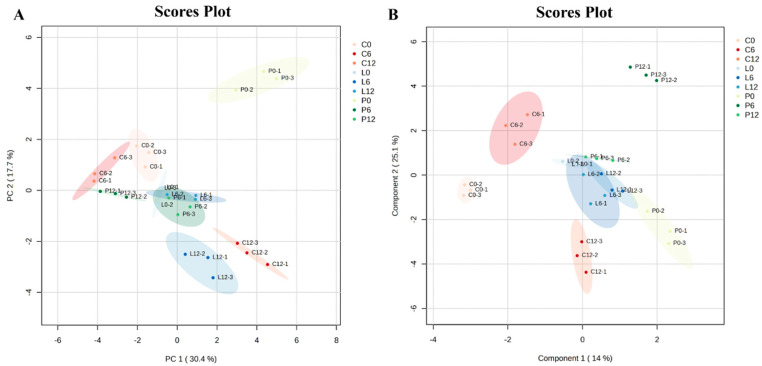
Multivariate statistical analysis of the mass spectral data set for large yellow croaker samples. (**A**) PCA score plot showing the spatial distribution of nine sample groups. (**B**) PLS-DA score plot presenting the distribution of 9 sample groups. Each group is color-coded and plotted according to its score on the two components.

Principal component analysis (PCA) was employed to visually differentiate large yellow croaker samples preserved by different methods over a storage period of 0 to 12 days. As shown in Figure 1A, the short distances between the three parallel samples reflect the reproducibility of the data. The plot in Figure 1A shows a slight overlap between 0 and 6 days in Group C, followed by a significant diagonal separation by day 12, indicating marked changes in volatile components over time for this group. Similarly, Group L showed comparable volatile compound profiles on days 0 and 6, whereas notable differences were observed on day 12, indicating significant changes in volatile composition over the storage period. Although partial clustering trends were observed in the PCA plot, considerable overlap among six of the nine groups suggests that the separation of volatile profiles was not distinct. The PCA plot shows that the first two principal components explain 48.1% of the total variance, with PC1 and PC2 accounting for 30.4% individually in Figure 1A. Although the variance explained by the first component is relatively low, the separation trend among the various groups is evident.

Xu et al. (2021) [24] reported that the PCA model is influenced by experimental factors, such as instrumentation, and to mitigate these issues, partial least squares discriminant analysis (PLS-DA) was applied. Figure 1B presents the PLS-DA score plot, where partial group separation can be observed. Although some overlap exists among at least three groups, the relatively compact clustering within individual groups suggests acceptable repeatability of the PLS-DA model. Compared to PCA, the PLS-DA score plot emphasizes classification by maximizing the groups. In the present analysis, PC1 and PC2 of the PLS-DA model accounted for 14% and 26.1% of the variation, respectively, suggesting a trade-off between variance explanation and class discrimination.

**Figure 2:**
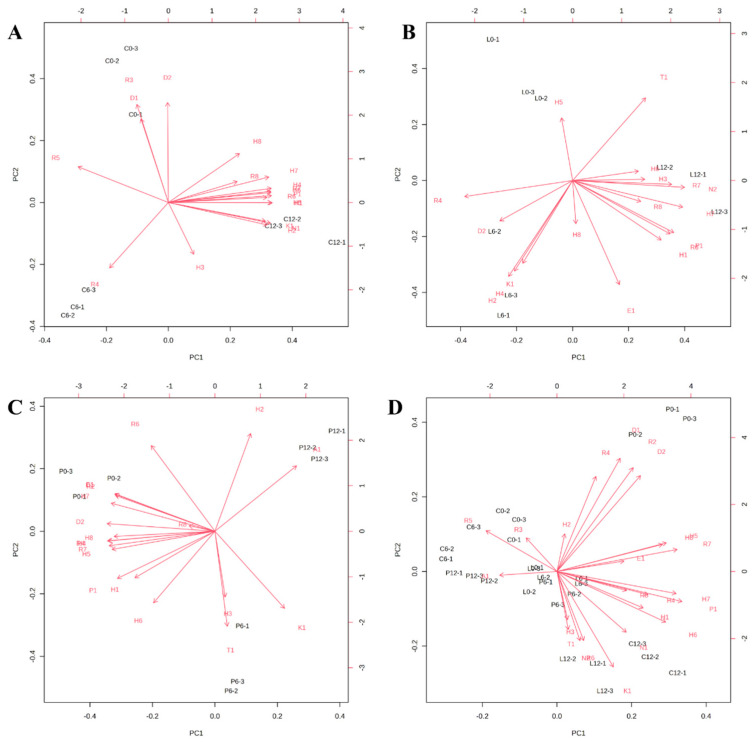
Loading plot of the variables in the cold storage group (**A**), slurry ice group (**B**), crushed ice group (**C**) and all samples (**D**) based on the PCA model.

To further analyze the association between each volatile compound and the principal components, we conducted a loading analysis based on the PCA results. The loading plots for the different treatment groups are displayed in Figure 2, where greater distance from the zero point indicates a stronger contribution to the model. In Figure 2A, triethyl phosphate, pentyl phosphate, and benzoic acid 2-methylpropyl ester show significant contributions to both PC1 and PC2. In Figure 2B, heptane, benzeneacetic acid, and benzene,1,3-dimethyl are located at relatively greater distances from the origin, suggesting potential relevance to group separation. Figure 2C displays dimethyl phthalate, butylated hydroxytoluene, and benzene,1,3-dimethyl as compounds with comparatively prominent loading values. However, it should be emphasized that PCA is an unsupervised method and does not inherently provide information on the statistical significance or odor activity of individual compounds. Therefore, while these compounds may appear to influence sample distribution in the PCA space, further statistical validation is required to confirm their roles in odor differentiation. When data from all treatment groups across different storage durations were combined into a unified PCA loading plot, aldehydes such as benzaldehyde and nonanal, along with hydrocarbons like toluene, n-dodecane, and limonene, emerged as consistent contributors to the variance in volatile profiles. These compounds may represent key odor-active markers in large yellow croaker during storage. Nonetheless, their definitive impact should be confirmed through complementary analyses such as VIP scoring, odor threshold evaluation, or sensory correlation studies. Additionally, when the experimental groups for the three treatments on different days were combined into a single loading plot, aldehydes (benzaldehyde and nonanal) and hydrocarbons (toluene, n-dodecane, and limonene) emerged as the key factors influencing the volatile profile of large yellow croaker.

### 3.3. Identification of Characteristic Volatile Compounds

The ANOVA statistical results of 24 volatiles with high contribution rates are listed in Table 2. In the C group, benzene (H2), caryophyllene (H6), dodecane (H4), hexadecane (H8), naphthalene (H3), pentadecane (H7), sulfurous acid (H8), and toluene (H1) showed positive correlations with the storage time of large yellow croaker. Conversely, nonanal displayed a negative correlation. In the L group, pentadecane (H7), sulfurous acid, 2-ethylhexyl tridecyl ester (R8), toluene (H1), caryophyllene (H6), and naphthalene (H3) were positively correlated with storage time, while benzene,1,3-dimethyl (H2), dodecane (H4), hexadecane (H8), and nonanal (D2) exhibited negative correlations. In the P group, benzene showed a negative correlation with storage time, whereas pentadecane (H7), sulfurous acid, 2-ethylhexyl tridecyl ester (R8), toluene (H1), caryophyllene (H6), naphthalene (H3), dodecane (H4), hexadecane (H8), and nonanal (D2) were positively correlated. To further explore the relationship between large yellow croaker groups and characteristic volatiles, we calculated the correlation coefficients (r) between groups and the relative content of characteristic volatiles. The relative content was determined by first calculating the peak area of a specific ion fragment, followed by using a relative quantification method with 2,4,6-trimethylpyridine as an internal standard. All volatiles with |r| > 0.4 are listed in Table 2, which showed that the selected characteristic volatile compounds have stronger correlation coefficients than other volatile compounds.

### 3.4. Identification of Potential Characteristic Volatile Compounds

**Figure 3:**
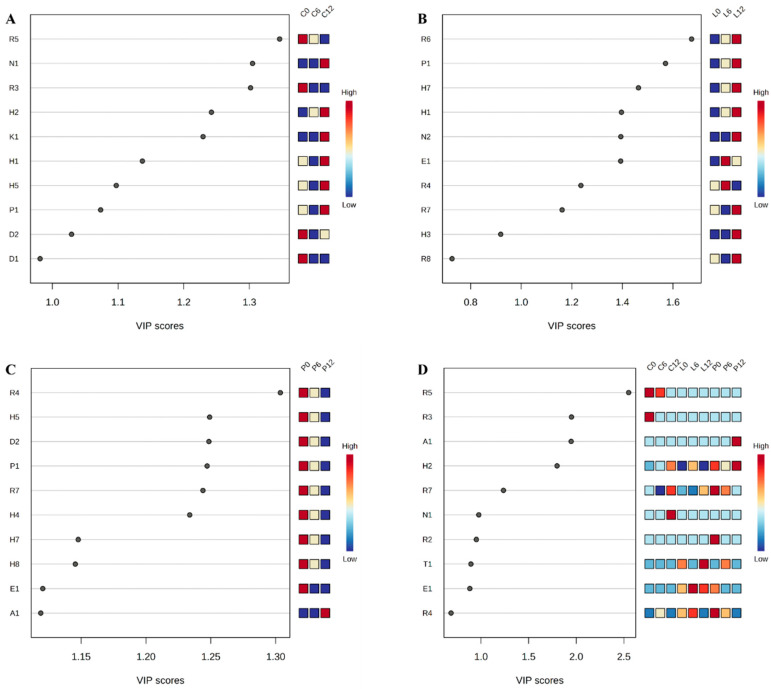
Top 10 volatile compounds that contribute significantly to the separation of these nine groups by the PLS-DA approach. The contribution can be quantified by VIP scores listed on the *x* axis. The color represents the relative concentration of each volatile compound from different sample groups. (**A**–**C**) show the VIP values for the three treatments C, P, and L, respectively, and (**D**) shows the overall VIP value.

To assess the discriminatory power of each compound, variable importance in projection (VIP) scores derived from the PLS-DA model were employed. Only compounds with VIP scores greater than one were considered significant contributors to group separation and were thus selected as key volatile markers. The VIP (variable importance in projection) scores, which provide the volatile compounds of three experimental groups in Figure 3A–C (VIP > 1), highlight the top 10 volatile compounds contributing to the separation in the PLS-DA model, with VIP values greater than one (as explained in Table 1). Among these volatile compounds, 2-methylpropanoate, triethyl phosphate, and 1,2-propanediol are the top three significantly different volatile compounds, as illustrated in Figure 3D.

Based on the analysis of these volatile compounds, we found that the three treatment methods significantly influenced the composition of volatiles in large yellow croaker during storage. These changes primarily involved nitrogen compounds, aldehydes, ketones, esters, phenols, alcohols, ethers, hydrocarbons, and other compounds. Among these, only volatiles with a VIP (variable importance in projection) score above one were deemed significant contributors to the fish’s volatile profile. From the loadings and VIP plots, specific compounds emerged as key differentiators among the treatment groups. In Group C, 1,3-dimethylbenzene (H2), dodecane (H4), nonanal (D2), and toluene (H1) were the primary contributors to variations in volatile profiles. For Group L, pentadecane (H7) and 2,5-cyclohexadiene-1,4-dione, 2-(1,1-dimethylethyl)-5-(2-methyl-2-propen-1-yl) (R7) were identified as the main distinguishing compounds. Meanwhile, Group P was characterized by nonanal (D2), 2,5-cyclohexadiene-1,4-dione, 2-(1,1-dimethylethyl)-5-(2-methyl-2-propen-1-yl) (R7), n-dodecane (H4), n-pentadecane (H7), and n-hexadecane (H8). Although 10 characteristic volatile compounds were identified in this study, VIP plot analysis suggested that only benzene,1,3-dimethyl (H2) and 2,5-cyclohexadiene-1,4-dione, 2-(1,1-dimethylethyl)-5-(2-methyl-2-propen-1-yl) (R7) were key in distinguishing different groups of large yellow croaker samples.

### 3.5. Differences in Volatile Compounds of Large Yellow Croaker Under Different Storage Conditions

**Figure 4:**
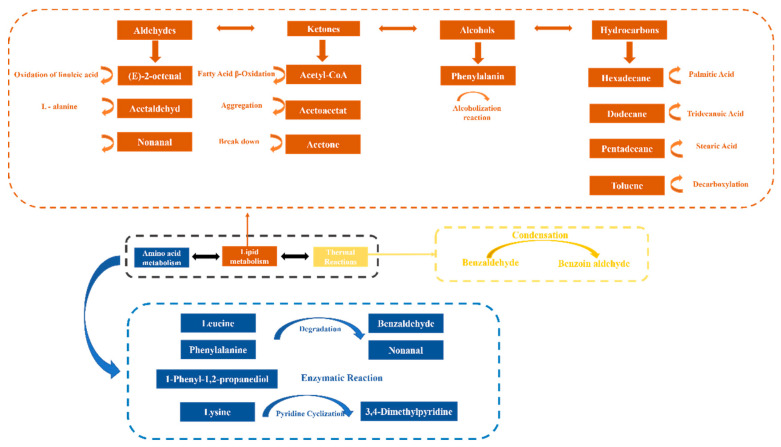
Brief scheme representation of the formation pathways of characteristic volatile compounds identified in large yellow croaker.

Several studies have analyzed the volatiles in fish during storage [25]. While these studies mainly focused on identifying various volatiles and their correlation with quality attributes, the mechanisms driving these transformations remain insufficiently understood. Here, three metabolic pathways were summarized that contributed to the generation of characteristic volatiles of large yellow croaker. Although the present study did not directly investigate the biochemical mechanisms underlying volatile formation, this schematic (Figure 4) provides a theoretical framework for understanding the possible origins of these compounds. 

#### 3.5.1. Thermal Reaction Volatile Compounds Pathway

Although previous studies have proposed that thermal reactions may contribute to the formation of specific volatile compounds in fish muscle, their relevance under cool storage conditions remains questionable. In the volatiles during the storage of large yellow croaker, only 3,4-dimethylpyrimidine and benzoin aldehyde were detected, while the other glycolytic compounds were notably absent. This suggests that changes in heat treatment might have rendered these compounds non-significant. In addition, 3,4-dimethylpyrimidine and benzoin aldehyde, volatiles extracted from nitrogen-containing compounds during storage of large yellow croaker, were identified as contributing a ‘smoky’ and ‘almondy’ aroma, respectively [26]. In this study, while the thermal reaction pathway cannot be overlooked, its contribution to the overall volatile profile is minimal (Table 2). The reason for this is that the volatiles produced by large yellow croaker during storage are mainly aliphatic volatile compounds, with relatively high levels of alcohols and aldehydes (Table 2). The reason why thermal reactions are not important in this study is because it is performed under cool storage. Given that the major volatiles identified were aliphatic alcohols and aldehydes (Table 2), lipid oxidation and amino acid degradation are more likely to be the predominant pathways contributing to the odor changes observed.

#### 3.5.2. Lipid Oxidation Pathway

The lipid oxidation pathway during storage of the large yellow croaker is shown in Figure 4. During the storage of large yellow croaker, fat degradation and oxidation are the primary factors influencing its odor, leading to the production of characteristic volatile compounds [8]. In the present study, the primary compounds associated with fat degradation and oxidation included aldehydes, ketones, and alcohols, including benzaldehyde (D1), nonanal (D2), toluene (H1), Benzene,1,3-dimethyl (H2), naphthalene (H3), dodecane (H4), caryophyllene (H6), pentadecane (H7), hexadecane (H8), and 2-(formyloxy)-1-phenyl(K1). Among these, nonanal was the predominant aldehyde during the storage of large yellow croaker and was a key factor affecting its odor. Nonanal, primarily derived from the oxidation of unsaturated fatty acids like EPA and DHA, showed high VIP (variable importance in projection) values in PLS-DA analysis, making it a reliable marker for assessing the quality of large yellow croaker. Lipid oxidation and degradation are the primary causes of rancid volatiles in fish during storage [27]. Additionally, benzaldehyde, which imparts an almond-like aroma, is generated through a deacidification reaction. Specifically, lipid oxidation triggers the volatile compounds of linoleic acid, resulting in benzaldehyde as a final product (Figure 4). Given its low odor threshold, benzaldehyde significantly influences the overall odor profile of the fish [28].

**Figure 5:**
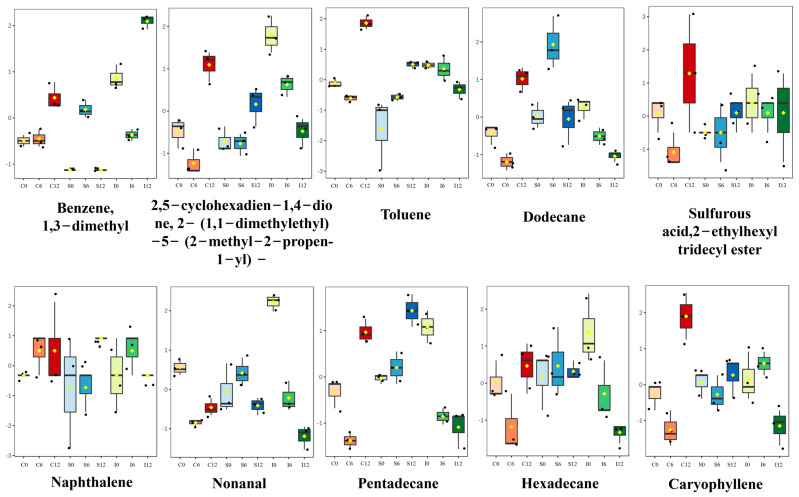
Box plots of 12 example volatile compounds showing significant differences during storage of these nine groups. Normalized intensities are presented on the *y*-axis.

Additionally, fat oxidation is accompanied by the production of small organic molecules, such as ketones, hydrocarbons, and alcohols, which significantly contribute to the odor of meat [29]. In this study, acetone was identified as a key ketone, primarily resulting from the oxidation of fatty acids (e.g., linoleic acid). This process produces peroxides that subsequently break down into acetophenone, contributing to a distinct sweet aroma. In addition, only 1,2-propanediol was detected among the alcohol compounds during storage, indicating relatively low levels of lipid degradation products. This suggests that the three treatments applied effectively slowed the oxidation of large yellow croaker. Box plot analysis (Figure 5) revealed that five hydrocarbons—toluene (H1), Benzene,1,3-dimethyl (H2), n-dodecane (H4), pentadecane (H7), and hexadecane (H8)—played a role in shaping the odor profile of large yellow croaker, primarily through lipid oxidation. Notably n-Dodecane (H4), pentadecane (H7), and hexadecane (H8) levels gradually decreased after 12 days of storage due to ongoing fat degradation and nutrient depletion (see Table 2), with these changes being less pronounced in the L-treated group. Additionally, free radicals and hydroperoxides generated during fat oxidation can serve as precursors or promoters for amino acid degradation [30].

#### 3.5.3. Amino Acid Degradation Pathway

The amino acid degradation pathway during storage of the large yellow croaker is shown in Figure 4. While changes in meat odor are often attributed to lipid oxidation, amino acid degradation also plays a significant role in the deterioration of fish during storage [31]. The analysis of the three treatment groups revealed several sulfur- and nitrogen-containing compounds with relatively small peak areas, such as 2-phenylethyl ester, pyridine, and 3,4-dimethylbenzene. These results suggest that the applied storage treatments—cold storage (CS), slurry ice (SI), and crushed ice (CI)—may have effectively suppressed amino acid degradation in large yellow croaker. According to Table 2, the volatile compounds also did not produce substances with an unpleasant odor. Typically, odors resulting from amino acid degradation stem from protein degradation, leading to the production of sulfur- and nitrogen-based compounds. This process involves enzymes breaking down proteins into various amino acids (Figure 4), which are then further decomposed through deamination, desulfurization, and decarboxylation reactions to yield organic acids, sulfur compounds, ammonia, amines, and hydrocarbons. Based on the changes in the relative content of volatiles observed (Table 2), it was evident that the compounds derived from amino acid degradation were present in minimal amounts across the three treatments during the storage of large yellow croaker. This suggests that lipid oxidation was the primary contributor to the characteristic volatile profile of large yellow croaker during storage, while the contribution of amino acid degradation was relatively minor.

Certain compounds have been widely reported to play key roles in the odor deterioration of large yellow croaker fillets. As illustrated in Figure 4, the potential formation pathways of the characteristic volatile compounds identified in this study are summarized based on the relevant literature and the exploration of volatile substances in this experiment. These pathways primarily include amino acid degradation, lipid oxidation, and thermal reactions, which are commonly associated with spoilage and processing-induced changes in fish products.

## 4. Conclusions

In this study, HS-SPME-GC-MS was used to analyze the differences in characteristic volatile compounds of large yellow croaker fillets stored under three different conditions: a cold storage group, a slurry ice group, and a crushed ice group. Several common volatile compounds were found in large yellow croaker, including toluene, 1,3-dimethylbenzene, nonanal, dodecane, caryophyllene, 2,5-cyclohexadiene-1,4-dione, 2-(1,1-dimethylethyl)-5-(2-methyl-2-propen-1-yl), butylated hydroxytoluene, hexadecane, and sulfurous acid, 2-ethylhexyl tridecyl ester. By combining the results from the loading plot and VIP score analysis, 10 characteristic volatile compounds were identified as biomarkers. Moreover, the pathways and transformations of each volatile were thoroughly investigated, employing multivariate analysis across the three storage methods at 0, 6, and 12 days. The study identified three primary pathways involved in volatile compound formation—thermal reactions, lipid oxidation, and amino acid degradation. Overall, among the three proposed pathways—lipid oxidation, amino acid degradation, and thermal reactions—lipid oxidation was identified as the dominant contributor to the formation of key volatiles in large yellow croaker during cold storage, particularly aldehydes and alcohols such as nonanal and benzaldehyde. In contrast, amino acid degradation played a minor role, as indicated by the low abundance of nitrogen- and sulfur-containing compounds and the absence of typical off-odor metabolites. Thermal reactions contributed minimally under refrigeration conditions, with only a few products such as 3,4-dimethylpyrimidine. These findings underscore lipid oxidation as the primary pathway shaping the odor profile under the tested storage conditions. In conclusion, the SI treatment was found to be the most effective method for maintaining the quality of large yellow croaker fillets during storage. Its ability to mitigate lipid oxidation and stabilize key volatiles makes it a promising strategy for extending shelf life and preserving sensory attributes in commercial aquatic product storage. These findings provide new insights into developing storage strategies for aquatic products in commercial applications.

## Figures and Tables

**Table 1:** Volatile compounds of large yellow croaker identified through HS-SPME-GC-MS during storage.

Super Class	R.T. (min)	Volatile Compounds	Abbreviation	CAS	Formula
Nitrogen compound	8.971	Nicotinic acid, 2-phenylethyl ester	N1	1000308-36-9	C_14_H_13_NO_2_
	9.794	Pyridine, 3,4-dimethyl	N2	000583-58-4	C_7_H_9_N
Aldehydes	10.549	Benzaldehyde	D1	000100-52-7	C_7_H_6_O
	13.521	Nonanal	D2	000124-19-6	C_9_H_18_O
Hydrocarbons	6.02	Toluene	H1	000108-88-3	C_7_H8
	8.447	Benzene,1,3-dimethyl	H2	000108-38-3	C_8_H_10_
	15.083	Naphthalene	H3	000091-20-3	C_10_H_8_
	15.251	Dodecane	H4	000112-40-3	C_12_H_26_
	16.111	Benzene,1,3-bis(1,1-dimethylethyl)-	H5	001014-60-4	C_14_H_22_
	18.176	Caryophyllene	H6	000087-44-5	C_15_H_24_
	18.789	Pentadecane	H7	000629-62-9	C_15_H_32_
	20.467	Hexadecane	H8	000544-76-3	C_16_H_34_
Ketones	10.539	Ethanone, 2-(formyloxy)-1-phenyl	K1	055153-12-3	C_9_H_8_O_3_
Ethers	11.991	Heptane,1,1′-oxybis-	E1	000629-64-1	C_14_H_30_O
Alcohols	12.127	1,2-Propanediol,1-phenyl	A1	001855-09-0	C_9_H_12_O_2_
Phenols	19.000	Butylated Hydroxytoluene	P1	000128-37-0	C_15_H_24_O
Esters	13.181	Benzeneacetic acid,	T1	056143-21-6	C_10_H_12_O_3_
Others	13.175	Benzene,(1-methoxypropyl)	R2	059588-12-4	C_10_H_14_O
	14.009	Triethyl phosphate	R3	000078-40-0	C_6_H_15_O_4_P
	14.014	Phosphoric acid, diethyl pentyl ester	R4	020195-08-8	C_9_H_21_O_4_P
	17.070	Benzoic acid, 2-methylpropyl ester	R5	000120-50-3	C_11_H_14_O_2_
	18.438	Dimethyl phthalate	R6	000131-11-3	C_10_H_10_O_4_
	18.695	2,5-cyclohexadiene-1,4-dione,	R7	1000396-22-4	C_14_H_18_O_2_
	20.520	Sulfurous acid, 2-ethylhexyl tridecyl ester	R8	1000309-19-6	C_21_H_44_O_3_S

**Table 2:** Changes in the relative peak area of volatile compounds during storage (estimated means ± standard error) and correlation analysis with storage days.

Volatile Compounds	C_0_	C_6_	C_12_	L_0_	L_6_	L_12_	P_0_	P_6_	P_12_
Toluene	0.54 ^ab^ ± 0.023	0.46 ^a^ ± 0.02	0.86 ^b^ ± 0.035	0.28 ^a^ ± 0.24	0.47 ^a^ ± 0.02	0.64 ^a^ ± 0.020	0.64 ^b^ ± 0.020	0.62 ^b^ ± 0.06	0.51 ^a^ ± 0.04
Benzene,1,3-dimethyl	0.06 ^b^ ± 0.010	0.06 ^a^ ± 0.015	0.13 ^a^ ± 0.023	0.01 ^a^ ± 0.001	0.11 ^b^ ± 0.015	n.d.	0.17 ^c^ ± 0.020	0.07 ^a^ ± 0.01	0.26 ^b^ ± 0.01
Nicotinic acid, 2-phenylethyl ester	n.d.	n.d.	0.16 ^b^ ± 0.015	n.d.	n.d.	n.d.	n.d.	n.d.	n.d.
Ethanone, 2-(formyloxy)-1-phenyl	n.d.	n.d.	0.04 ^a^ ± 0.015	0.02 ^b^ ± 0.005	0.04 ^b^ ± 0.005	0.02 ^a^ ± 0.005	n.d.	0.03 ^b^ ± 0.005	0.02 ^a^ ± 0.005
Benzaldehyde	0.03 ^a^ ± 0.026	n.d.	n.d.	n.d.	n.d.	n.d.	0.09 ^b^ ± 0.011	n.d.	n.d.
Heptane,1,1-oxybis-	n.d.	n.d.	n.d.	0.02 ^a^ ± 0.015	0.16 ^b^ ± 0.015	0.15 ^b^ ± 0.015	0.13 ^b^ ± 0.015	n.d.	n.d.
1,2-Propanediol,1-phenyl	n.d.	n.d.	n.d.	n.d.	n.d.	n.d.	n.d.	n.d.	0.05 ^b^ ± 0.01
Benzene,(1-methoxypropyl)	n.d.	n.d.	n.d.	n.d.	n.d.	n.d.	0.08 ^b^ ± 0.002	n.d.	n.d.
Benzeneacetic acid, alpha.-methoxy-,methyl ester	n.d.	n.d.	n.d.	0.02 ± 0.005	n.d.	0.03 ± 0.005	n.d.	0.02 ± 0.01	n.d.
Pyridine, 3,4-dimethyl	n.d.	n.d.	n.d.	n.d.	n.d.	0.04 ± 0.010	n.d.	n.d.	n.d.
Nonanal	0.17 ^a^ ± 0.015	0.08 ^a^ ± 0.005	0.10 ^a^ ± 0.015	0.13 ^a^ ± 0.043	0.16 ^c^ ± 0.025	0.11 ^b^ ± 0.015	0.29 ^b^ ± 0.02	0.12 ^b^ ± 0.03	0.05 ^a^ ± 0.02
Triethyl phosphate	0.05 ^a^ ± 0.005	n.d.	n.d.	n.d.	n.d.	n.d.	n.d.	0.00	0.00
Phosphoric acid, diethyl pentyl ester	n.d.	0.02 ^a^ ± 0.001	n.d.	0.02 ^a^	0.02 ^a^ ± 0.005	n.d.	0.04 ^a^ ± 0.002	0.02 ^a^ ± 0.005	n.d.
Naphthalene	0.03 ^a^ ± 0.002	0.04 ^a^ ± 0.005	0.03 ^a^ ± 0.011	0.03 ^a^ ± 0.005	0.03 ^a^ ± 0.005	0.04 ^a^ ± 0.002	0.03 ^a^ ± 0.01	0.04 ^a^ ± 0.01	0.03 ^a^ ± 0.002
Dodecane	0.11 ^a^ ± 0.011	0.08 ^a^ ± 0.01	0.18 ^b^ ± 0.015	0.13 ^a^ ± 0.015	0.22 ^b^ ± 0.030	0.13 ^a^ ± 0.026	0.14 ^a^ ± 0.01	0.11 ^a^ ± 0.01	0.09 ^a^ ± 0.01
Benzene,1,3-bis(1,1-dimethylethyl)	0.09 ^a^ ± 0.01	0.06 ^a^ ± 0.152	0.18 ^b^ ± 0.081	0.12 ^ab^ ± 0.081	0.08 ^a^ ± 0.020	0.1 ^a^ ± 0.020	0.20 ^b^ ± 0.01	0.13 ^ab^ ± 0.03	0.06 ^a^ ± 0.01
Benzoic acid,2-methylpropyl ester	0.02 ± 0.005	0.02 ± 0.005	n.d.	n.d.	n.d.	n.d.	n.d.	n.d.	n.d.
Caryophyllene	0.07 ^ab^ ± 0.011	0.04 ^a^ ± 0.152	0.14 ^b^ ± 0.011	0.08 ^ab^ ± 0.011	0.07 ^ab^ ± 0.015	0.09 ^ab^ ± 0.017	0.09 ^ab^ ± 0.02	0.10 ^ab^ ± 0.01	0.05 ^a^ ± 0.01
Dimethyl phthalate	0.02 ^a^ ± 0.005	n.d.	0.06 ^a^ ± 0.026	0.24 ^b^ ± 0.160	2.24 ^c^ ± 0.056	4.63 ^d^ ± 0.070	0.06 ^a^ ± 0.01	n.d.	0.03 ^a^ ± 0.01
2,5-cyclohexadiene-1,4dione, 2-(1,1-dimethylethyl)-5-(2-methyl-2-propen-1-yl)	0.14 ^a^ ± 0.020	0.10 ^a^ ± 0.173	0.23 ^b^ ± 0.021	0.13 ^a^ ± 0.017	0.13 ^a^ ± 0.015	0.18 ^a^ ± 0.026	0.27 ^b^ ± 0.03	0.21 ^ab^ ± 0.01	0.14 ^a^ ± 0.02
Pentadecane	0.17 ^a^ ± 0.017	0.11 ^a^ ± 0.01	0.24 ^b^ ± 0.015	0.19 ^ab^ ± 0.005	0.20 ^ab^ ± 0.020	0.27 ^b^ ± 0.02	0.25 ^b^ ± 0.020	0.14 ^a^ ± 0.01	0.13 ^a^ ± 0.02
Butylated Hydroxytoluene	14.19 ^a^ ± 0.352	11.63 ^a^ ± 0.854	21.27 ^c^ ± 0.113	14.03 ^a^ ± 0.643	14.88 ^b^ ± 0.135	16.19 ^b^ ± 0.110	17.93 ^b^ ± 0.14	16.75 ^c^ ± 0.21	13.08 ^a^ ± 0.20
Hexadecane	0.05 ^ab^ ± 0.011	0.03 ^a^ ± 0.001	0.07 ^b^ ± 0.015	0.06 ^ab^ ± 0.001	0.07 ^b^ ± 0.020	0.06 ^ab^ ± 0.005	0.09 ^c^ ± 0.02	0.05 ^ab^ ± 0.02	0.03 ^a^ ± 0.01
Sulfurous acid, 2-ethylhexyl tridecyl ester	0.05 ^a^ ± 0.005	0.04 ^a^ ± 0.005	0.07 ^a^ ± 0.02	0.05 ^a^ ± 0.001	0.05 ^a^ ± 0.010	0.06 ^a^ ± 0.005	0.06 ^a^ ± 0.01	0.06 ^a^ ± 0.01	0.06 ^a^ ± 0.01

Letters a–d in the same column indicate a significant difference (*p* < 0.05). Data are expressed as estimated means ± standard error (*n* = 3). n.d. represents undetected substances.

## Data Availability

The original contributions presented in the study are included in the article/Supplementary Material, further inquiries can be directed to the corresponding authors.

## Supplementary Materials

The following supporting information can be downloaded at: https://www.mdpi.com/article/10.3390/foods14122063/s1, Figure S1: Counts vs. Collection time (min).

## Abbreviations

Cold storage group (CS): Five fish were placed directly into pre-cooled (2 °C) expanded polypropylene (EPP) (430 × 210 × 270 mm^3^) boxes (21 L) stored in an artificial climate chamber at 2 °C. Slurry ice group (SI): Five fish were evenly immersed into seawater slurry ice in the EPP box and stored in an artificial climate chamber at 2 °C. Crushed ice group (CI): Five fish and freshwater crushed ice were evenly distributed in the EPP box and stored in an artificial climate chamber at 2 °C.
