# Supplementary material for: A Comparative Study Based on HS-SPME-GC-MS of Volatile Compounds in Large Yellow Croaker (Pseudosciaena crocea) During Varied Cold Storage Conditions
Source: Foods. 2025 Jun 11;14(12):2063. doi: 10.3390/foods14122063 (PMC12192311; doi:10.3390/foods14122063)
Supplement: Supplementary file 1 [file foods-14-02063-s001.zip › foods-3503473-supplementary/补充文件/C0 _Analysis-structure.template.pdf]

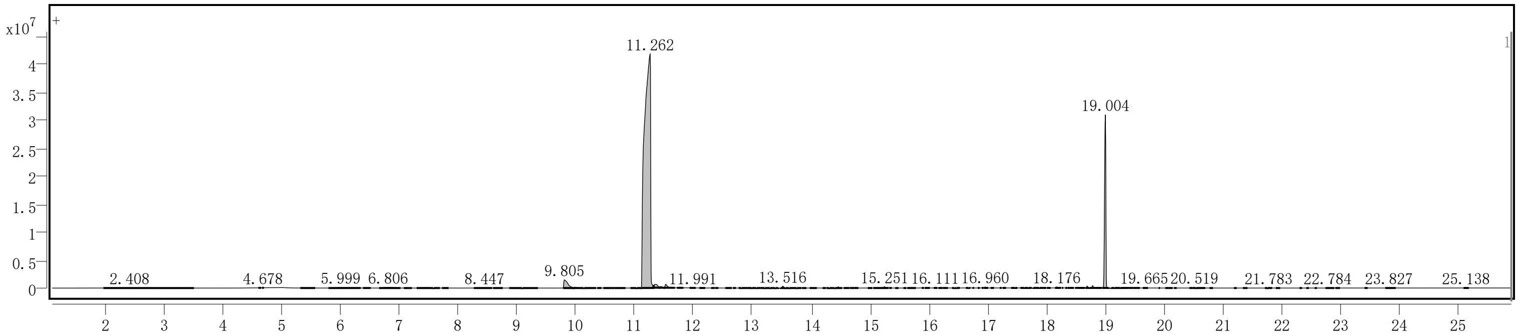

Chromatogram Peaks

Peak	Start	RT	End	Height	Area	Area %	SNR
1	1.965	2.408	3.503	21170	1034955	0.35	
2	4.599	4.636	4.647	10335	18544	0.01	
3	4.660	4.678	4.694	8769	9698	0.00	
4	5.312	5.401	5.566	8035	61241	0.02	
5	5.791	5.999	6.340	93561	1817588	0.61	
6	6.379	6.476	6.510	5664	23085	0.01	
7	6.653	6.806	7.016	122575	1621696	0.54	
8	7.074	7.152	7.210	10037	51109	0.02	
9	7.291	7.472	7.577	87843	782813	0.26	
10	7.577	7.650	7.686	22570	81143	0.03	
11	7.722	7.776	7.834	4555	17262	0.01	
12	8.264	8.447	8.573	29270	262212	0.09	
13	8.589	8.641	8.756	4233	20631	0.01	
14	8.866	8.976	9.087	29051	171467	0.06	
15	9.087	9.176	9.353	31691	206078	0.07	
16	9.784	9.805	9.957	1466766	6972606	2.34	
17	9.957	9.978	10.135	155837	596428	0.20	
18	10.135	10.193	10.343	18788	142113	0.05	
19	10.362	10.381	10.434	2968	7621	0.00	
20	10.466	10.554	10.601	28135	131420	0.04	
21	10.601	10.617	10.654	6739	13474	0.00	
22	10.654	10.759	10.843	39362	187146	0.06	
23	10.927	10.989	11.031	42872	180064	0.06	
24	11.031	11.079	11.110	60660	166489	0.06	
25	11.110	11.262	11.320	41751693	297940178	100.00	
26	11.320	11.351	11.419	596197	2619219	0.88	
27	11.419	11.440	11.503	203880	722845	0.24	
28	11.503	11.529	11.684	614014	1833098	0.62	
29	11.718	11.744	11.828	23455	95465	0.03	
30	11.933	11.991	12.036	42576	117707	0.04	
31	12.101	12.132	12.199	13545	45774	0.02	
32	12.299	12.316	12.361	5311	11496	0.00	
33	12.369	12.389	12.423	6769	10674	0.00	
34	12.541	12.573	12.650	16177	50040	0.02	
35	12.671	12.693	12.721	7979	13365	0.00	
36	12.767	12.793	12.814	30923	53076	0.02	
37	12.814	12.856	12.918	48807	153573	0.05	
38	12.918	12.955	12.992	36593	88030	0.03	
39	12.992	13.018	13.070	38269	99931	0.03	
40	13.070	13.107	13.139	26229	58902	0.02	
41	13.139	13.175	13.212	25362	59248	0.02	
42	13.212	13.243	13.301	29062	96418	0.03	
43	13.301	13.348	13.390	37973	87820	0.03	
44	13.390	13.422	13.443	27860	50650	0.02	
45	13.485	13.516	13.563	316356	602151	0.20	
46	13.563	13.600	13.626	22447	62990	0.02	
47	13.626	13.668	13.757	54673	152636	0.05	
48	13.757	13.778	13.836	15527	36922	0.01	
49	13.845	13.878	13.920	12873	25896	0.01	
50	13.978	14.004	14.097	69084	185146	0.06	
51	14.194	14.219	14.250	10687	17669	0.01	
52	14.260	14.281	14.308	19693	32960	0.01	

Chromatogram Peaks

Peak	Start	RT	End	Height	Area	Area %	SNR
53	14.308	14.334	14.402	13776	50707	0.02	
54	14.402	14.460	14.528	206489	427225	0.14	
55	14.554	14.580	14.606	5762	10736	0.00	
56	14.606	14.633	14.653	4037	5144	0.00	
57	14.660	14.690	14.727	20207	42553	0.01	
58	14.727	14.758	14.804	97275	153313	0.05	
59	14.963	15.015	15.042	20001	52629	0.02	
60	15.059	15.083	15.209	33606	92610	0.03	
61	15.209	15.251	15.319	234071	356106	0.12	
62	15.330	15.356	15.371	6944	9204	0.00	
63	15.430	15.477	15.487	8632	12347	0.00	
64	15.571	15.597	15.618	7182	11772	0.00	
65	15.635	15.676	15.780	50827	139628	0.05	
66	15.881	15.896	15.919	15905	16456	0.01	
67	15.933	15.980	16.018	41948	82298	0.03	
68	16.086	16.111	16.148	135716	185171	0.06	
69	16.166	16.184	16.237	13222	22782	0.01	
70	16.242	16.273	16.326	11133	24411	0.01	
71	16.397	16.410	16.436	15384	19690	0.01	
72	16.436	16.452	16.467	11404	12377	0.00	
73	16.467	16.494	16.529	22061	35327	0.01	
74	16.633	16.656	16.682	80988	112901	0.04	
75	16.688	16.703	16.708	5562	3110	0.00	
76	16.745	16.750	16.771	8816	6363	0.00	
77	16.834	16.855	16.866	12148	13300	0.00	
78	16.903	16.923	16.934	11176	10656	0.00	
79	16.939	16.960	17.011	156166	226386	0.08	
80	17.052	17.070	17.109	37569	60724	0.02	
81	17.208	17.222	17.239	9826	10396	0.00	
82	17.255	17.269	17.315	11658	21364	0.01	
83	17.401	17.458	17.469	22751	63706	0.02	
84	17.469	17.484	17.505	34802	41107	0.01	
85	17.560	17.584	17.626	57651	98104	0.03	
86	17.626	17.678	17.694	23805	52613	0.02	
87	17.694	17.715	17.752	29731	52475	0.02	
88	17.778	17.799	17.883	136334	274446	0.09	
89	17.883	17.904	17.951	17553	38525	0.01	
90	17.951	17.972	17.998	13782	20614	0.01	
91	18.010	18.029	18.073	10241	22564	0.01	
92	18.148	18.176	18.249	184312	247726	0.08	
93	18.266	18.276	18.286	7584	4572	0.00	
94	18.325	18.339	18.357	11649	10533	0.00	
95	18.364	18.391	18.407	7708	8864	0.00	
96	18.407	18.438	18.468	51650	72213	0.02	
97	18.491	18.512	18.543	19254	32044	0.01	
98	18.543	18.580	18.656	68352	121459	0.04	
99	18.662	18.695	18.732	325676	448634	0.15	
100	18.732	18.753	18.769	91584	120101	0.04	
101	18.769	18.790	18.842	407168	543701	0.18	
102	18.842	18.910	18.947	15151	55348	0.02	
103	18.952	19.004	19.062	30941339	47675138	16.00	
104	19.104	19.130	19.157	30367	55758	0.02	
105	19.157	19.198	19.256	96684	232613	0.08	
106	19.256	19.277	19.398	28972	133061	0.04	
107	19.398	19.419	19.476	15243	24358	0.01	
108	19.476	19.560	19.590	12785	36945	0.01	
109	19.640	19.665	19.730	64105	81775	0.03	
110	19.911	19.922	19.934	4342	2863	0.00	
111	20.023	20.053	20.079	12857	18839	0.01	
112	20.079	20.121	20.147	19828	27320	0.01	
113	20.173	20.194	20.216	26900	33188	0.01	
114	20.432	20.467	20.488	121603	136354	0.05	
115	20.488	20.519	20.546	133619	164309	0.06	
116	20.546	20.567	20.703	66394	192040	0.06	
117	20.777	20.803	20.835	11135	16163	0.01	
118	21.190	21.217	21.238	5420	7486	0.00	
119	21.343	21.363	21.379	3977	4211	0.00	
120	21.379	21.400	21.421	12418	14011	0.00	
121	21.717	21.746	21.762	39419	61917	0.02	
122	21.762	21.783	21.840	38774	68147	0.02	
123	21.903	21.930	21.977	7609	12755	0.00	
124	22.307	22.312	22.349	2975	2969	0.00	
125	22.422	22.449	22.464	6347	7758	0.00	
126	22.564	22.585	22.601	4197	5266	0.00	
127	22.747	22.784	22.898	26818	88759	0.03	
128	22.916	22.931	22.993	4485	8594	0.00	
129	23.413	23.444	23.465	5788	8962	0.00	
130	23.764	23.827	23.942	17796	64286	0.02	
131	25.097	25.138	25.185	8215	20646	0.01	
