# Supplementary material for: A Comparative Study Based on HS-SPME-GC-MS of Volatile Compounds in Large Yellow Croaker (Pseudosciaena crocea) During Varied Cold Storage Conditions
Source: Foods. 2025 Jun 11;14(12):2063. doi: 10.3390/foods14122063 (PMC12192311; doi:10.3390/foods14122063)
Supplement: Supplementary file 1 [file foods-14-02063-s001.zip › foods-3503473-supplementary/补充文件/C12 _Analysis-structure.template.pdf]

Analysis Report

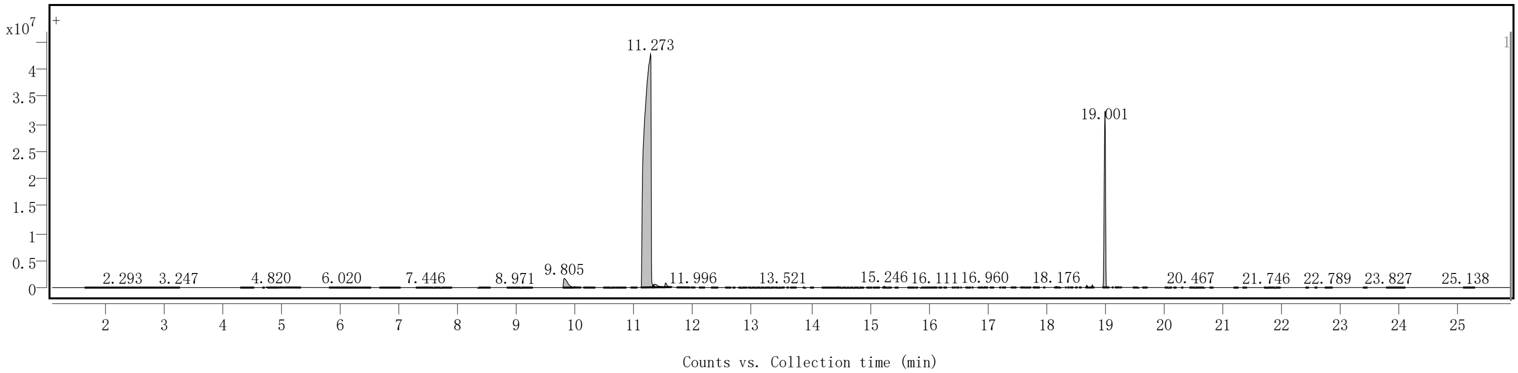

Chromatogram Peaks

Peak	Star	RT	End	Height	Area	Area %	SNR
1	1.643	2.293	3.194	40413	2058422	0.62	
2	3.195	3.247	3.268	6135	13363	0.00	
3	4.292	4.379	4.530	7722	52287	0.02	
4	4.668	4.704	4.710	5542	10244	0.00	
5	4.746	4.820	4.924	143402	922883	0.28	
6	4.924	4.945	4.961	62579	117726	0.04	
7	4.961	4.987	5.019	63604	198529	0.06	
8	5.019	5.035	5.050	65209	102535	0.03	
9	5.050	5.092	5.326	60627	488566	0.15	
10	5.801	6.020	6.350	77053	1473053	0.45	
11	6.351	6.492	6.518	8175	54067	0.02	
12	6.660	6.822	7.021	20336	239654	0.07	
13	7.279	7.446	7.587	88769	840027	0.25	
14	7.587	7.661	7.708	23724	96507	0.03	
15	7.708	7.802	7.897	8498	53211	0.02	
16	8.342	8.442	8.552	23942	191404	0.06	
17	8.827	8.971	9.071	57717	407973	0.12	
18	9.071	9.113	9.270	12273	84499	0.03	
19	9.779	9.805	9.957	1685754	8206665	2.49	
20	9.957	9.978	10.091	170579	555451	0.17	
21	10.130	10.193	10.334	16907	125681	0.04	
22	10.467	10.539	10.602	48948	237254	0.07	
23	10.602	10.623	10.680	12606	31122	0.01	
24	10.681	10.754	10.861	19203	91188	0.03	
25	10.933	10.942	11.049	28333	79833	0.02	
26	11.107	11.273	11.320	42736647	329766316	100.00	
27	11.320	11.346	11.503	567091	2983073	0.90	
28	11.503	11.529	11.634	774459	1829869	0.55	
29	11.716	11.739	11.944	30514	150892	0.05	
30	11.954	11.996	12.035	58190	116034	0.04	
31	12.096	12.132	12.199	15121	49215	0.01	
32	12.300	12.331	12.425	13527	45288	0.01	
33	12.542	12.573	12.645	11263	29745	0.01	
34	12.667	12.688	12.716	5774	8381	0.00	
35	12.765	12.793	12.824	21477	43056	0.01	
36	12.824	12.856	12.919	15044	46178	0.01	
37	12.929	12.960	12.987	23973	52459	0.02	
38	12.987	13.023	13.071	38475	105089	0.03	
39	13.071	13.107	13.144	27280	60225	0.02	
40	13.144	13.181	13.212	29085	64076	0.02	
41	13.212	13.259	13.312	34692	120371	0.04	
42	13.312	13.343	13.385	11860	26648	0.01	
43	13.390	13.422	13.448	32880	59441	0.02	
44	13.448	13.474	13.490	23617	39721	0.01	
45	13.490	13.521	13.553	120545	205289	0.06	
46	13.584	13.600	13.626	11340	14778	0.00	
47	13.646	13.668	13.757	45337	95517	0.03	
48	13.864	13.878	13.904	7256	11512	0.00	
49	13.988	14.019	14.052	10076	14974	0.00	
50	14.184	14.224	14.250	9329	20638	0.01	
51	14.250	14.339	14.397	15776	68877	0.02	
52	14.397	14.460	14.528	188794	414815	0.13	

Chromatogram Peaks

Peak	Start	RT	End	Height	Area	Area %	SNR
53	14.528	14.580	14.606	9038	24521	0.01	
54	14.606	14.643	14.659	9311	17788	0.01	
55	14.659	14.685	14.727	11318	33065	0.01	
56	14.727	14.759	14.821	57630	109471	0.03	
57	14.821	14.884	14.906	6456	17985	0.01	
58	14.944	15.021	15.044	14470	48341	0.01	
59	15.054	15.084	15.175	47610	113846	0.03	
60	15.215	15.246	15.309	260693	387474	0.12	
61	15.309	15.356	15.377	7543	16191	0.00	
62	15.425	15.471	15.492	10061	13192	0.00	
63	15.644	15.676	15.774	35162	104355	0.03	
64	15.782	15.812	15.821	3951	5040	0.00	
65	15.875	15.980	16.080	52368	190563	0.06	
66	16.080	16.111	16.152	148258	220439	0.07	
67	16.169	16.184	16.236	14584	24577	0.01	
68	16.256	16.273	16.315	11820	21679	0.01	
69	16.398	16.415	16.431	16324	17046	0.01	
70	16.436	16.457	16.465	11177	11283	0.00	
71	16.474	16.494	16.526	14884	23385	0.01	
72	16.544	16.551	16.567	7398	5272	0.00	
73	16.630	16.656	16.677	105753	141786	0.04	
74	16.677	16.703	16.722	28251	48205	0.01	
75	16.736	16.750	16.766	15867	13444	0.00	
76	16.840	16.855	16.871	21418	22232	0.01	
77	16.871	16.923	16.934	24308	40102	0.01	
78	16.934	16.960	17.014	163969	265228	0.08	
79	17.044	17.070	17.117	23677	56210	0.02	
80	17.205	17.217	17.239	8718	11592	0.00	
81	17.253	17.296	17.319	11667	26348	0.01	
82	17.401	17.453	17.499	21614	73958	0.02	
83	17.563	17.584	17.657	53872	106410	0.03	
84	17.657	17.678	17.694	24360	32692	0.01	
85	17.694	17.715	17.746	27403	44236	0.01	
86	17.778	17.799	17.895	139144	284979	0.09	
87	17.954	17.972	17.989	9180	12382	0.00	
88	18.140	18.176	18.244	206545	290500	0.09	
89	18.327	18.339	18.349	8954	7065	0.00	
90	18.363	18.386	18.400	7500	8841	0.00	
91	18.410	18.438	18.466	86646	121198	0.04	
92	18.496	18.517	18.538	22005	31231	0.01	
93	18.561	18.580	18.606	69879	84665	0.03	
94	18.669	18.695	18.732	404332	527692	0.16	
95	18.732	18.753	18.769	79701	103141	0.03	
96	18.769	18.790	18.825	465400	568340	0.17	
97	18.961	19.001	19.078	30140375	51238872	15.54	
98	19.120	19.136	19.149	18391	16055	0.00	
99	19.173	19.199	19.256	88442	161104	0.05	
100	19.261	19.277	19.290	16011	13321	0.00	
101	19.478	19.492	19.534	6697	13798	0.00	
102	19.534	19.555	19.571	11054	13831	0.00	
103	19.640	19.660	19.728	73325	98898	0.03	
104	20.020	20.053	20.090	13703	22356	0.01	
105	20.090	20.121	20.144	18828	27113	0.01	
106	20.168	20.195	20.222	29877	39093	0.01	
107	20.299	20.310	20.336	5365	6373	0.00	
108	20.441	20.467	20.493	140263	155003	0.05	
109	20.494	20.520	20.540	98824	126623	0.04	
110	20.540	20.572	20.693	75259	218166	0.07	
111	20.782	20.797	20.850	12830	21930	0.01	
112	21.190	21.217	21.277	7838	12718	0.00	
113	21.343	21.363	21.379	10927	13787	0.00	
114	21.379	21.395	21.421	15460	18692	0.01	
115	21.708	21.746	21.762	48412	83648	0.03	
116	21.762	21.783	21.898	43903	121986	0.04	
117	21.898	21.930	21.993	9665	24916	0.01	
118	22.413	22.454	22.474	6195	8835	0.00	
119	22.569	22.580	22.616	5049	7128	0.00	
120	22.742	22.789	22.878	33122	91926	0.03	
121	23.392	23.439	23.471	4711	9408	0.00	
122	23.785	23.827	24.116	19588	115761	0.04	
123	25.096	25.138	25.300	8536	45325	0.01	
