# Supplementary material for: A Comparative Study Based on HS-SPME-GC-MS of Volatile Compounds in Large Yellow Croaker (Pseudosciaena crocea) During Varied Cold Storage Conditions
Source: Foods. 2025 Jun 11;14(12):2063. doi: 10.3390/foods14122063 (PMC12192311; doi:10.3390/foods14122063)
Supplement: Supplementary file 1 [file foods-14-02063-s001.zip › foods-3503473-supplementary/补充文件/C6 _Analysis-structure.template.pdf]

Analysis Report

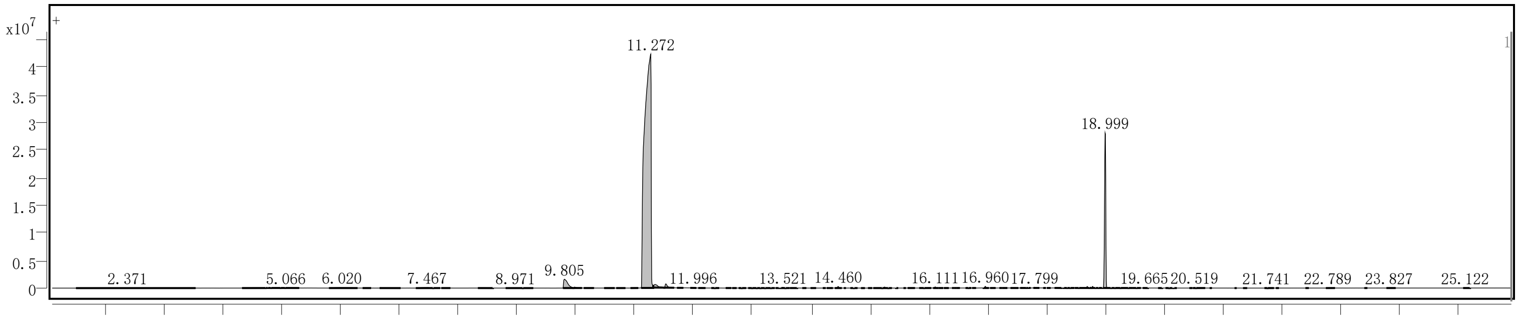

Chromatogram Peaks

Peak	Steat	RT	End	Height	Area	Area %	SNR
1	1.496	2.371	3.533	21905	1276780	0.40	
2	4.321	4.720	4.725	31786	340572	0.11	
3	4.725	4.746	4.762	22595	33222	0.01	
4	4.762	4.809	4.814	27553	67569	0.02	
5	4.814	4.971	4.977	44314	331347	0.10	
6	4.977	5.034	5.055	51584	201893	0.06	
7	5.055	5.066	5.294	52292	298142	0.09	
8	5.799	6.020	6.286	89423	1668218	0.52	
9	6.371	6.486	6.517	7689	39378	0.01	
10	6.661	6.811	7.018	33238	434131	0.14	
11	7.276	7.467	7.571	90749	826990	0.26	
12	7.571	7.655	7.691	25283	94478	0.03	
13	7.710	7.771	7.865	6070	26094	0.01	
14	8.332	8.447	8.588	23447	195172	0.06	
15	8.803	8.971	9.097	35057	244048	0.08	
16	9.097	9.191	9.275	11485	78271	0.02	
17	9.779	9.805	9.957	1591633	7749211	2.42	
18	9.957	9.972	10.103	165351	550298	0.17	
19	10.133	10.177	10.308	13949	99150	0.03	
20	10.478	10.554	10.659	15558	81987	0.03	
21	10.685	10.753	10.843	23008	106995	0.03	
22	10.927	10.942	11.061	29074	106137	0.03	
23	11.110	11.272	11.314	42285040	320162828	100.00	
24	11.314	11.346	11.503	563291	3059649	0.96	
25	11.503	11.529	11.672	690117	1854087	0.58	
26	11.718	11.739	11.828	27140	86540	0.03	
27	11.944	11.996	12.049	52500	118331	0.04	
28	12.081	12.127	12.204	22919	67575	0.02	
29	12.301	12.321	12.363	10384	24802	0.01	
30	12.363	12.394	12.436	14872	31883	0.01	
31	12.546	12.572	12.644	9535	22574	0.01	
32	12.656	12.693	12.733	7745	19332	0.01	
33	12.767	12.793	12.819	32298	55078	0.02	
34	12.819	12.840	12.895	15298	38948	0.01	
35	12.930	12.960	12.997	31644	76598	0.02	
36	12.997	13.018	13.076	25198	66087	0.02	
37	13.076	13.107	13.139	16672	33200	0.01	
38	13.139	13.180	13.217	30583	63948	0.02	
39	13.217	13.264	13.317	18285	64608	0.02	
40	13.317	13.348	13.382	16537	29499	0.01	
41	13.395	13.422	13.453	18914	31835	0.01	
42	13.485	13.521	13.563	144791	265744	0.08	
43	13.563	13.605	13.634	16780	39455	0.01	
44	13.638	13.668	13.762	52247	118663	0.04	
45	13.849	13.883	13.920	12891	23539	0.01	
46	13.989	14.014	14.085	35738	86827	0.03	
47	14.197	14.224	14.248	7025	10986	0.00	
48	14.264	14.287	14.308	6554	9032	0.00	
49	14.308	14.334	14.370	12142	26432	0.01	
50	14.403	14.460	14.521	277067	590702	0.18	
51	14.557	14.570	14.593	3853	3822	0.00	
52	14.667	14.690	14.722	11284	20673	0.01	

Chromatogram Peaks

峰	起始	RT	终止	峰高	面积	面积 %	SNR
53	14.732	14.758	14.790	41928	65580	0.02	
54	14.847	14.895	14.916	5362	12520	0.00	
55	14.966	15.020	15.039	12003	32031	0.01	
56	15.057	15.083	15.209	32346	88137	0.03	
57	15.209	15.251	15.314	185954	292166	0.09	
58	15.314	15.356	15.375	7944	16903	0.01	
59	15.445	15.466	15.482	4484	4880	0.00	
60	15.570	15.597	15.613	4994	6600	0.00	
61	15.641	15.676	15.767	54455	131503	0.04	
62	15.838	15.891	15.922	27944	55460	0.02	
63	15.922	15.980	16.043	49733	131516	0.04	
64	16.074	16.111	16.168	209140	305954	0.10	
65	16.168	16.189	16.247	16329	31484	0.01	
66	16.258	16.273	16.341	8930	25431	0.01	
67	16.396	16.415	16.431	15058	16990	0.01	
68	16.431	16.452	16.467	10372	11696	0.00	
69	16.467	16.493	16.530	17485	31469	0.01	
70	16.626	16.656	16.677	71812	99539	0.03	
71	16.677	16.703	16.724	21961	41845	0.01	
72	16.737	16.750	16.805	6703	16344	0.01	
73	16.934	16.960	17.024	290521	440470	0.14	
74	17.043	17.070	17.138	33359	67099	0.02	
75	17.202	17.227	17.248	11391	19503	0.01	
76	17.248	17.295	17.337	16294	49551	0.02	
77	17.390	17.458	17.525	25170	108747	0.03	
78	17.554	17.584	17.662	55807	121660	0.04	
79	17.662	17.678	17.694	16416	16515	0.01	
80	17.694	17.715	17.734	15704	21095	0.01	
81	17.779	17.799	17.883	132859	248838	0.08	
82	17.946	17.972	17.992	12201	16129	0.01	
83	18.009	18.061	18.074	8189	17134	0.01	
84	18.139	18.176	18.260	112468	189954	0.06	
85	18.297	18.339	18.354	17460	23880	0.01	
86	18.354	18.391	18.407	11281	23790	0.01	
87	18.407	18.438	18.480	49480	74034	0.02	
88	18.485	18.517	18.554	20389	43567	0.01	
89	18.554	18.580	18.664	54999	111137	0.03	
90	18.664	18.695	18.732	272524	380394	0.12	
91	18.732	18.753	18.768	157385	196589	0.06	
92	18.768	18.789	18.837	280652	390882	0.12	
93	18.837	18.868	18.889	15921	33884	0.01	
94	18.889	18.910	18.947	15774	36158	0.01	
95	18.962	18.999	19.099	28062044	41168401	12.86	
96	19.104	19.130	19.167	29267	63809	0.02	
97	19.167	19.198	19.261	97355	240959	0.08	
98	19.261	19.277	19.398	33065	136367	0.04	
99	19.398	19.418	19.471	15057	32915	0.01	
100	19.471	19.523	19.534	10891	27004	0.01	
101	19.534	19.560	19.607	14978	27909	0.01	
102	19.639	19.665	19.728	64916	89094	0.03	
103	19.901	19.932	19.969	5276	10426	0.00	
104	20.027	20.053	20.089	16227	23452	0.01	
105	20.089	20.121	20.147	26066	35584	0.01	
106	20.173	20.194	20.219	40450	52151	0.02	
107	20.432	20.467	20.488	71646	79243	0.02	
108	20.493	20.519	20.546	95617	119871	0.04	
109	20.546	20.567	20.703	64845	166066	0.05	
110	20.777	20.802	20.817	8008	9545	0.00	
111	21.201	21.222	21.237	4077	5174	0.00	
112	21.345	21.395	21.416	12619	20733	0.01	
113	21.709	21.741	21.762	42100	68175	0.02	
114	21.762	21.783	21.872	37308	87145	0.03	
115	21.903	21.924	21.956	7011	11697	0.00	
116	22.406	22.454	22.464	6402	9514	0.00	
117	22.753	22.789	22.910	27605	82579	0.03	
118	23.408	23.434	23.460	5859	8412	0.00	
119	23.783	23.827	23.942	14200	55237	0.02	
120	25.088	25.122	25.206	8203	28272	0.01	
