# Supplementary material for: A Comparative Study Based on HS-SPME-GC-MS of Volatile Compounds in Large Yellow Croaker (Pseudosciaena crocea) During Varied Cold Storage Conditions
Source: Foods. 2025 Jun 11;14(12):2063. doi: 10.3390/foods14122063 (PMC12192311; doi:10.3390/foods14122063)
Supplement: Supplementary file 1 [file foods-14-02063-s001.zip › foods-3503473-supplementary/补充文件/L0 Analysis-structure.template.pdf]

Analysis Report

Agilent Trusted Answers

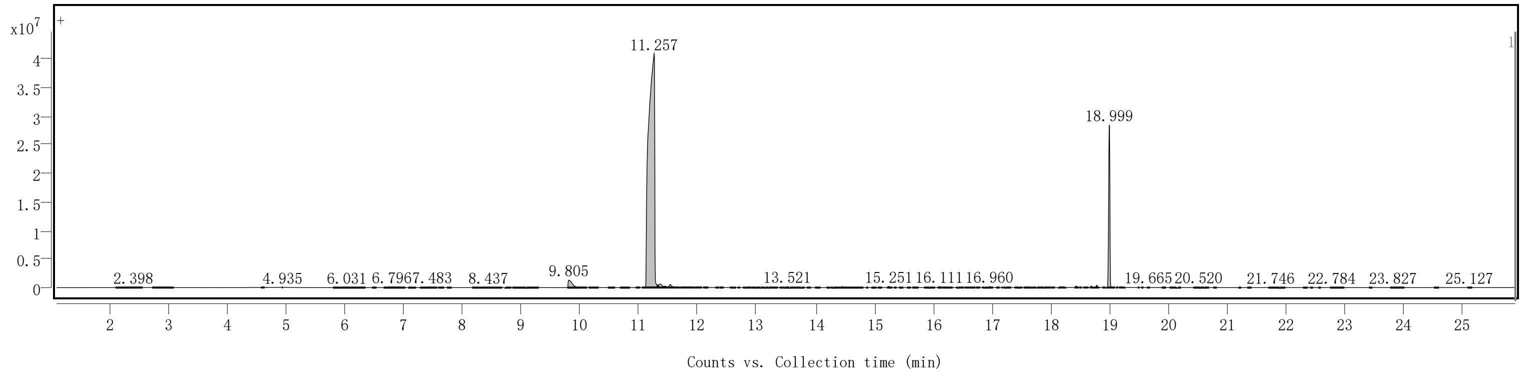

Chromatogram Peaks

Peak	Start	RT	End	Height	Area	Area %	SNR
1	2.095	2.398	2.555	10042	152926	0.05	
2	2.718	2.770	3.084	5138	85872	0.03	
3	4.568	4.605	4.631	7424	11203	0.00	
4	4.924	4.935	4.940	6637	4155	0.00	
5	5.795	6.031	6.345	63268	1229509	0.43	
6	6.455	6.487	6.534	3939	10335	0.00	
7	6.656	6.796	7.027	131872	1773330	0.62	
8	7.074	7.158	7.207	10230	47123	0.02	
9	7.273	7.483	7.566	81680	728382	0.25	
10	7.583	7.661	7.685	19684	62959	0.02	
11	7.732	7.776	7.813	5265	12639	0.00	
12	8.163	8.248	8.332	6317	41072	0.01	
13	8.332	8.437	8.610	32582	283835	0.10	
14	8.610	8.641	8.673	2692	6359	0.00	
15	8.725	8.777	8.827	4986	12743	0.00	
16	8.846	8.987	9.081	29654	192977	0.07	
17	9.081	9.191	9.295	38924	250146	0.09	
18	9.782	9.805	9.962	1309199	6401968	2.23	
19	9.962	9.983	10.114	133299	449359	0.16	
20	10.142	10.187	10.312	10828	65298	0.02	
21	10.470	10.539	10.593	14125	56614	0.02	
22	10.675	10.748	10.846	35670	168273	0.06	
23	10.944	10.974	11.020	51292	113940	0.04	
24	11.051	11.068	11.100	19932	31384	0.01	
25	11.100	11.257	11.320	40880261	286922096	100.00	
26	11.320	11.367	11.419	583144	2636277	0.92	
27	11.419	11.440	11.498	225998	805932	0.28	
28	11.498	11.529	11.697	551216	1880792	0.66	
29	11.697	11.739	11.944	33138	225270	0.08	
30	11.944	11.991	12.071	33793	122175	0.04	
31	12.090	12.127	12.179	14241	39795	0.01	
32	12.300	12.321	12.358	5532	10427	0.00	
33	12.368	12.394	12.442	5464	10434	0.00	
34	12.547	12.578	12.615	14149	31803	0.01	
35	12.615	12.625	12.652	9399	12391	0.00	
36	12.679	12.698	12.718	8468	10781	0.00	
37	12.770	12.793	12.814	20081	31612	0.01	
38	12.814	12.850	12.917	29004	94423	0.03	
39	12.920	12.955	12.987	35573	77891	0.03	
40	12.987	13.018	13.071	34014	92493	0.03	
41	13.071	13.113	13.144	23300	50726	0.02	
42	13.144	13.175	13.207	26668	55075	0.02	
43	13.207	13.249	13.363	26889	99172	0.03	
44	13.392	13.422	13.448	23597	43857	0.02	
45	13.448	13.474	13.485	19901	28871	0.01	
46	13.485	13.521	13.563	155008	296496	0.10	
47	13.563	13.600	13.637	17763	42866	0.01	
48	13.637	13.668	13.763	42906	113678	0.04	
49	13.763	13.773	13.821	9376	20981	0.01	
50	13.854	13.878	13.923	12060	20727	0.01	
51	13.995	14.025	14.087	24477	55846	0.02	
52	14.191	14.219	14.251	9010	14641	0.01	

Chromatogram Peaks

Peak	Start	RT	End	Height	Area	Area %	SNR
53	14.261	14.287	14.308	12352	18137	0.01	
54	14.308	14.329	14.402	12741	40177	0.01	
55	14.402	14.460	14.549	184641	407943	0.14	
56	14.549	14.575	14.607	7840	16454	0.01	
57	14.607	14.638	14.659	6393	11766	0.00	
58	14.659	14.685	14.732	14145	36981	0.01	
59	14.732	14.759	14.816	64286	105582	0.04	
60	14.865	14.890	14.898	2581	2345	0.00	
61	14.952	15.021	15.042	15553	47717	0.02	
62	15.063	15.084	15.136	15698	41420	0.01	
63	15.220	15.251	15.309	231857	341165	0.12	
64	15.335	15.351	15.382	7187	11249	0.00	
65	15.424	15.477	15.498	6607	13897	0.00	
66	15.561	15.602	15.623	3656	8458	0.00	
67	15.649	15.676	15.753	46786	100039	0.03	
68	15.849	15.896	15.943	14049	35815	0.01	
69	15.943	15.980	16.038	39708	90688	0.03	
70	16.080	16.111	16.158	201145	288309	0.10	
71	16.164	16.190	16.242	19360	38781	0.01	
72	16.242	16.305	16.336	7712	21967	0.01	
73	16.395	16.415	16.431	16478	18992	0.01	
74	16.431	16.452	16.473	15044	15125	0.01	
75	16.473	16.488	16.529	9024	19386	0.01	
76	16.541	16.551	16.572	6891	7314	0.00	
77	16.572	16.598	16.623	5391	8474	0.00	
78	16.626	16.656	16.724	82842	154294	0.05	
79	16.734	16.751	16.777	14550	18653	0.01	
80	16.777	16.787	16.804	4451	4116	0.00	
81	16.838	16.855	16.882	18220	22257	0.01	
82	16.882	16.923	16.934	10449	20963	0.01	
83	16.934	16.960	17.023	181026	280929	0.10	
84	17.077	17.091	17.136	12389	17142	0.01	
85	17.166	17.175	17.189	6826	4850	0.00	
86	17.199	17.222	17.243	11826	18375	0.01	
87	17.243	17.269	17.330	13423	40759	0.01	
88	17.391	17.458	17.469	23247	66125	0.02	
89	17.469	17.484	17.522	23255	32184	0.01	
90	17.543	17.584	17.663	50629	116718	0.04	
91	17.663	17.678	17.694	21376	28799	0.01	
92	17.694	17.715	17.752	20391	42587	0.01	
93	17.773	17.799	17.888	138047	277981	0.10	
94	17.888	17.930	17.946	13228	28473	0.01	
95	17.946	17.972	18.002	14063	20100	0.01	
96	18.007	18.030	18.074	11524	25477	0.01	
97	18.140	18.176	18.255	162878	251434	0.09	
98	18.410	18.433	18.482	252425	350288	0.12	
99	18.494	18.517	18.534	18412	21801	0.01	
100	18.560	18.580	18.617	51228	63192	0.02	
101	18.617	18.638	18.647	7040	7623	0.00	
102	18.670	18.695	18.732	252396	348813	0.12	
103	18.732	18.753	18.763	87498	96690	0.03	
104	18.763	18.790	18.830	404305	520411	0.18	
105	18.884	18.910	18.936	6540	11798	0.00	
106	18.957	18.999	19.098	28504235	41989558	14.63	
107	19.117	19.130	19.153	16649	19334	0.01	
108	19.167	19.199	19.261	69178	140009	0.05	
109	19.485	19.492	19.513	4760	3693	0.00	
110	19.545	19.560	19.580	9612	10519	0.00	
111	19.644	19.665	19.691	63610	73722	0.03	
112	19.890	19.927	19.964	4233	9624	0.00	
113	20.027	20.053	20.095	13309	17966	0.01	
114	20.095	20.121	20.147	28008	39160	0.01	
115	20.168	20.195	20.220	27332	35656	0.01	
116	20.430	20.467	20.493	92513	106560	0.04	
117	20.493	20.520	20.546	101407	129355	0.05	
118	20.546	20.567	20.619	50849	104064	0.04	
119	20.619	20.635	20.698	14342	28399	0.01	
120	20.771	20.797	20.829	6884	12922	0.00	
121	21.198	21.217	21.243	4166	4960	0.00	
122	21.348	21.395	21.421	11759	19180	0.01	
123	21.707	21.746	21.903	34925	127089	0.04	
124	21.903	21.930	21.997	6384	15958	0.01	
125	22.297	22.307	22.375	2451	7298	0.00	
126	22.418	22.449	22.464	5333	6034	0.00	
127	22.556	22.580	22.599	3459	3565	0.00	
128	22.758	22.784	22.889	21625	65010	0.02	
129	22.889	22.989	22.998	3078	12704	0.00	
130	23.418	23.439	23.476	5152	8964	0.00	
131	23.781	23.827	23.948	11477	47239	0.02	
132	23.948	23.995	24.021	3654	8815	0.00	
133	24.524	24.566	24.608	5424	13707	0.00	
134	25.093	25.127	25.174	6977	17722	0.01	
