# Supplementary material for: A Comparative Study Based on HS-SPME-GC-MS of Volatile Compounds in Large Yellow Croaker (Pseudosciaena crocea) During Varied Cold Storage Conditions
Source: Foods. 2025 Jun 11;14(12):2063. doi: 10.3390/foods14122063 (PMC12192311; doi:10.3390/foods14122063)
Supplement: Supplementary file 1 [file foods-14-02063-s001.zip › foods-3503473-supplementary/补充文件/L12 _Analysis-structure.template.pdf]

# Analysis Report

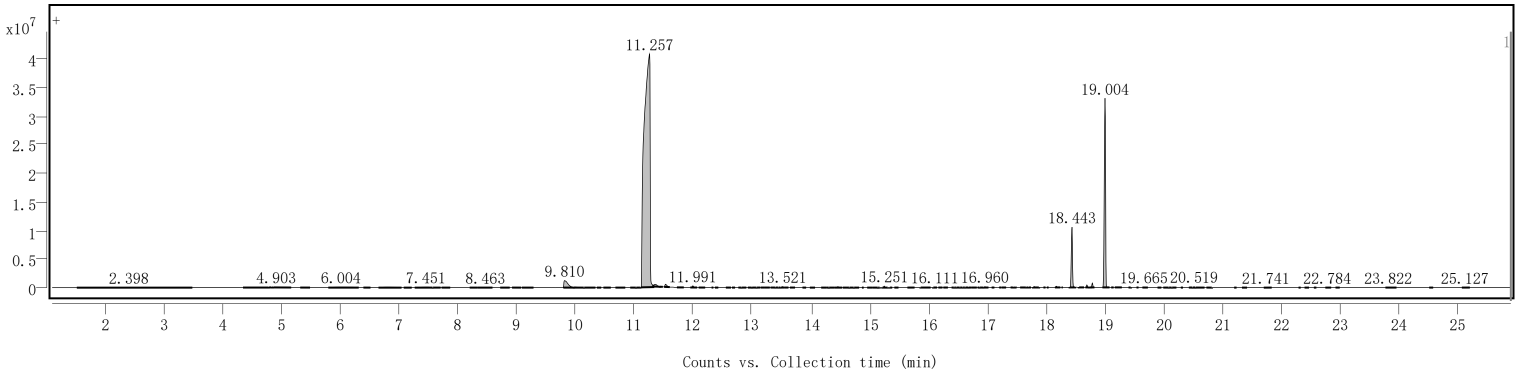


*Chromatogram Peaks*

| Peak | Start | RT | End | Height | Area | Area % | SNR |
|---|---|---|---|---|---|---|---|
| 1 | 1.512 | 2.398 | 3.477 | 20970 | 1230237 | 0.44 | |
| 2 | 4.339 | 4.772 | 4.777 | 61443 | 755672 | 0.27 | |
| 3 | 4.777 | 4.819 | 4.825 | 37534 | 84470 | 0.03 | |
| 4 | 4.825 | 4.830 | 4.851 | 42855 | 59603 | 0.02 | |
| 5 | 4.851 | 4.903 | 5.159 | 49579 | 560544 | 0.20 | |
| 6 | 5.312 | 5.407 | 5.479 | 7135 | 30041 | 0.01 | |
| 7 | 5.790 | 6.004 | 6.308 | 112552 | 2080306 | 0.74 | |
| 8 | 6.387 | 6.476 | 6.508 | 5879 | 24359 | 0.01 | |
| 9 | 6.644 | 6.785 | 7.037 | 92774 | 1251086 | 0.44 | |
| 10 | 7.073 | 7.168 | 7.210 | 7917 | 37292 | 0.01 | |
| 11 | 7.260 | 7.451 | 7.566 | 94413 | 843838 | 0.30 | |
| 12 | 7.566 | 7.661 | 7.702 | 28641 | 122028 | 0.04 | |
| 13 | 7.716 | 7.786 | 7.864 | 7218 | 30679 | 0.01 | |
| 14 | 8.195 | 8.279 | 8.332 | 4194 | 19067 | 0.01 | |
| 15 | 8.332 | 8.463 | 8.583 | 33686 | 286396 | 0.10 | |
| 16 | 8.714 | 8.772 | 8.877 | 10124 | 60897 | 0.02 | |
| 17 | 8.916 | 8.987 | 9.069 | 19880 | 88765 | 0.03 | |
| 18 | 9.081 | 9.191 | 9.281 | 28950 | 168977 | 0.06 | |
| 19 | 9.789 | 9.810 | 9.967 | 1230639 | 5936596 | 2.10 | |
| 20 | 9.967 | 9.983 | 10.140 | 132828 | 491697 | 0.17 | |
| 21 | 10.140 | 10.208 | 10.336 | 14529 | 100854 | 0.04 | |
| 22 | 10.359 | 10.397 | 10.434 | 2813 | 6291 | 0.00 | |
| 23 | 10.470 | 10.533 | 10.596 | 15593 | 71114 | 0.03 | |
| 24 | 10.675 | 10.764 | 10.843 | 16321 | 84098 | 0.03 | |
| 25 | 10.926 | 10.974 | 11.026 | 49009 | 164868 | 0.06 | |
| 26 | 11.026 | 11.068 | 11.099 | 40274 | 114703 | 0.04 | |
| 27 | 11.110 | 11.257 | 11.320 | 40797801 | 282475974 | 100.00 | |
| 28 | 11.320 | 11.351 | 11.430 | 394442 | 1533617 | 0.54 | |
| 29 | 11.430 | 11.440 | 11.482 | 81827 | 148202 | 0.05 | |
| 30 | 11.503 | 11.529 | 11.600 | 469289 | 1129121 | 0.40 | |
| 31 | 11.721 | 11.744 | 11.838 | 22868 | 94531 | 0.03 | |
| 32 | 11.949 | 11.991 | 12.065 | 280247 | 539217 | 0.19 | |
| 33 | 12.091 | 12.127 | 12.202 | 17509 | 54136 | 0.02 | |
| 34 | 12.310 | 12.326 | 12.341 | 3835 | 3717 | 0.00 | |
| 35 | 12.368 | 12.394 | 12.426 | 8891 | 14664 | 0.01 | |
| 36 | 12.553 | 12.578 | 12.641 | 12316 | 32783 | 0.01 | |
| 37 | 12.658 | 12.688 | 12.710 | 6079 | 8683 | 0.00 | |
| 38 | 12.766 | 12.793 | 12.819 | 42797 | 78816 | 0.03 | |
| 39 | 12.819 | 12.850 | 12.903 | 23051 | 67991 | 0.02 | |
| 40 | 12.928 | 12.955 | 12.997 | 50310 | 120994 | 0.04 | |
| 41 | 12.997 | 13.023 | 13.076 | 43561 | 97822 | 0.03 | |
| 42 | 13.076 | 13.107 | 13.137 | 30204 | 52528 | 0.02 | |
| 43 | 13.144 | 13.175 | 13.212 | 38047 | 72376 | 0.03 | |
| 44 | 13.212 | 13.264 | 13.302 | 30690 | 98335 | 0.03 | |
| 45 | 13.313 | 13.369 | 13.395 | 63269 | 142737 | 0.05 | |
| 46 | 13.395 | 13.416 | 13.448 | 31160 | 56290 | 0.02 | |
| 47 | 13.448 | 13.469 | 13.484 | 25477 | 36311 | 0.01 | |
| 48 | 13.484 | 13.521 | 13.558 | 174905 | 315940 | 0.11 | |
| 49 | 13.558 | 13.600 | 13.619 | 16933 | 33141 | 0.01 | |
| 50 | 13.645 | 13.668 | 13.731 | 49198 | 88061 | 0.03 | |
| 51 | 13.857 | 13.878 | 13.920 | 12667 | 21091 | 0.01 | |
| 52 | 13.983 | 14.019 | 14.071 | 11582 | 24176 | 0.01 | |

*Chromatogram Peaks*

| Peak | Start | RT | End | Height | Area | Area % | SNR |
|---|---|---|---|---|---|---|---|
| 53 | 14.175 | 14.218 | 14.260 | 9990 | 23748 | 0.01 | |
| 54 | 14.260 | 14.287 | 14.307 | 10461 | 19069 | 0.01 | |
| 55 | 14.307 | 14.339 | 14.407 | 13245 | 52381 | 0.02 | |
| 56 | 14.407 | 14.460 | 14.533 | 181772 | 389739 | 0.14 | |
| 57 | 14.543 | 14.580 | 14.606 | 7560 | 17592 | 0.01 | |
| 58 | 14.606 | 14.643 | 14.653 | 7870 | 12909 | 0.00 | |
| 59 | 14.653 | 14.685 | 14.732 | 15866 | 40866 | 0.01 | |
| 60 | 14.732 | 14.764 | 14.821 | 63478 | 113185 | 0.04 | |
| 61 | 14.868 | 14.879 | 14.899 | 3303 | 3062 | 0.00 | |
| 62 | 14.958 | 15.015 | 15.052 | 17824 | 59586 | 0.02 | |
| 63 | 15.052 | 15.083 | 15.141 | 45204 | 118568 | 0.04 | |
| 64 | 15.141 | 15.157 | 15.172 | 13761 | 20668 | 0.01 | |
| 65 | 15.214 | 15.251 | 15.309 | 253399 | 414268 | 0.15 | |
| 66 | 15.340 | 15.356 | 15.384 | 10845 | 17389 | 0.01 | |
| 67 | 15.420 | 15.471 | 15.491 | 12033 | 19917 | 0.01 | |
| 68 | 15.642 | 15.676 | 15.765 | 66507 | 159982 | 0.06 | |
| 69 | 15.864 | 15.980 | 16.037 | 45848 | 143433 | 0.05 | |
| 70 | 16.079 | 16.111 | 16.147 | 118640 | 168169 | 0.06 | |
| 71 | 16.169 | 16.189 | 16.240 | 24393 | 42935 | 0.02 | |
| 72 | 16.253 | 16.273 | 16.331 | 10670 | 29656 | 0.01 | |
| 73 | 16.394 | 16.410 | 16.436 | 21547 | 29218 | 0.01 | |
| 74 | 16.436 | 16.451 | 16.472 | 17552 | 22187 | 0.01 | |
| 75 | 16.472 | 16.499 | 16.535 | 14525 | 36443 | 0.01 | |
| 76 | 16.535 | 16.556 | 16.583 | 15186 | 22515 | 0.01 | |
| 77 | 16.583 | 16.604 | 16.624 | 9695 | 17744 | 0.01 | |
| 78 | 16.624 | 16.656 | 16.682 | 118801 | 174337 | 0.06 | |
| 79 | 16.682 | 16.703 | 16.729 | 36532 | 59014 | 0.02 | |
| 80 | 16.729 | 16.750 | 16.823 | 21927 | 48880 | 0.02 | |
| 81 | 16.835 | 16.855 | 16.871 | 24697 | 32800 | 0.01 | |
| 82 | 16.871 | 16.923 | 16.934 | 29207 | 64779 | 0.02 | |
| 83 | 16.934 | 16.960 | 17.022 | 152401 | 259685 | 0.09 | |
| 84 | 17.075 | 17.096 | 17.122 | 16242 | 24453 | 0.01 | |
| 85 | 17.203 | 17.222 | 17.246 | 10475 | 15399 | 0.01 | |
| 86 | 17.251 | 17.269 | 17.321 | 13288 | 32009 | 0.01 | |
| 87 | 17.397 | 17.421 | 17.442 | 21629 | 36861 | 0.01 | |
| 88 | 17.442 | 17.453 | 17.474 | 24032 | 33342 | 0.01 | |
| 89 | 17.474 | 17.484 | 17.500 | 22194 | 21881 | 0.01 | |
| 90 | 17.560 | 17.584 | 17.662 | 112709 | 197484 | 0.07 | |
| 91 | 17.662 | 17.683 | 17.699 | 23060 | 33074 | 0.01 | |
| 92 | 17.699 | 17.715 | 17.744 | 23535 | 33087 | 0.01 | |
| 93 | 17.778 | 17.799 | 17.883 | 167925 | 301807 | 0.11 | |
| 94 | 17.956 | 17.977 | 17.993 | 11846 | 16733 | 0.01 | |
| 95 | 18.014 | 18.029 | 18.045 | 12156 | 11506 | 0.00 | |
| 96 | 18.152 | 18.176 | 18.244 | 211752 | 271691 | 0.10 | |
| 97 | 18.265 | 18.281 | 18.285 | 5786 | 3740 | 0.00 | |
| 98 | 18.402 | 18.443 | 18.496 | 10540787 | 14949921 | 5.29 | |
| 99 | 18.559 | 18.580 | 18.648 | 77500 | 118748 | 0.04 | |
| 100 | 18.670 | 18.695 | 18.732 | 428783 | 558428 | 0.20 | |
| 101 | 18.732 | 18.753 | 18.768 | 80345 | 102773 | 0.04 | |
| 102 | 18.768 | 18.789 | 18.825 | 751481 | 897628 | 0.32 | |
| 103 | 18.960 | 19.004 | 19.081 | 33122601 | 51129099 | 18.10 | |
| 104 | 19.109 | 19.135 | 19.151 | 23789 | 32066 | 0.01 | |
| 105 | 19.170 | 19.198 | 19.290 | 91170 | 182072 | 0.06 | |
| 106 | 19.404 | 19.418 | 19.445 | 13588 | 13839 | 0.00 | |
| 107 | 19.540 | 19.555 | 19.575 | 14485 | 13677 | 0.00 | |
| 108 | 19.640 | 19.665 | 19.733 | 112628 | 152783 | 0.05 | |
| 109 | 19.897 | 19.927 | 19.963 | 6048 | 11787 | 0.00 | |
| 110 | 19.995 | 20.053 | 20.089 | 16708 | 33911 | 0.01 | |
| 111 | 20.089 | 20.116 | 20.142 | 24182 | 36185 | 0.01 | |
| 112 | 20.142 | 20.194 | 20.219 | 31019 | 50834 | 0.02 | |
| 113 | 20.294 | 20.320 | 20.331 | 4891 | 6226 | 0.00 | |
| 114 | 20.430 | 20.467 | 20.493 | 149955 | 182153 | 0.06 | |
| 115 | 20.493 | 20.519 | 20.540 | 156824 | 190472 | 0.07 | |
| 116 | 20.540 | 20.566 | 20.698 | 75014 | 207492 | 0.07 | |
| 117 | 20.729 | 20.797 | 20.823 | 9817 | 23534 | 0.01 | |
| 118 | 21.201 | 21.217 | 21.243 | 8618 | 10570 | 0.00 | |
| 119 | 21.334 | 21.395 | 21.421 | 12363 | 24762 | 0.01 | |
| 120 | 21.705 | 21.741 | 21.840 | 46237 | 142394 | 0.05 | |
| 121 | 22.296 | 22.317 | 22.333 | 4196 | 4567 | 0.00 | |
| 122 | 22.399 | 22.427 | 22.474 | 25901 | 40955 | 0.01 | |
| 123 | 22.557 | 22.585 | 22.600 | 5484 | 7222 | 0.00 | |
| 124 | 22.752 | 22.784 | 22.852 | 29307 | 70892 | 0.03 | |
| 125 | 22.925 | 22.967 | 22.997 | 3519 | 6200 | 0.00 | |
| 126 | 23.775 | 23.822 | 23.963 | 16956 | 67686 | 0.02 | |
| 127 | 24.519 | 24.571 | 24.587 | 4918 | 8920 | 0.00 | |
| 128 | 25.075 | 25.127 | 25.211 | 8957 | 32621 | 0.01 | |
