# Supplementary material for: A Comparative Study Based on HS-SPME-GC-MS of Volatile Compounds in Large Yellow Croaker (Pseudosciaena crocea) During Varied Cold Storage Conditions
Source: Foods. 2025 Jun 11;14(12):2063. doi: 10.3390/foods14122063 (PMC12192311; doi:10.3390/foods14122063)
Supplement: Supplementary file 1 [file foods-14-02063-s001.zip › foods-3503473-supplementary/补充文件/L6 _Analysis-structure.template.pdf]

Analysis Report

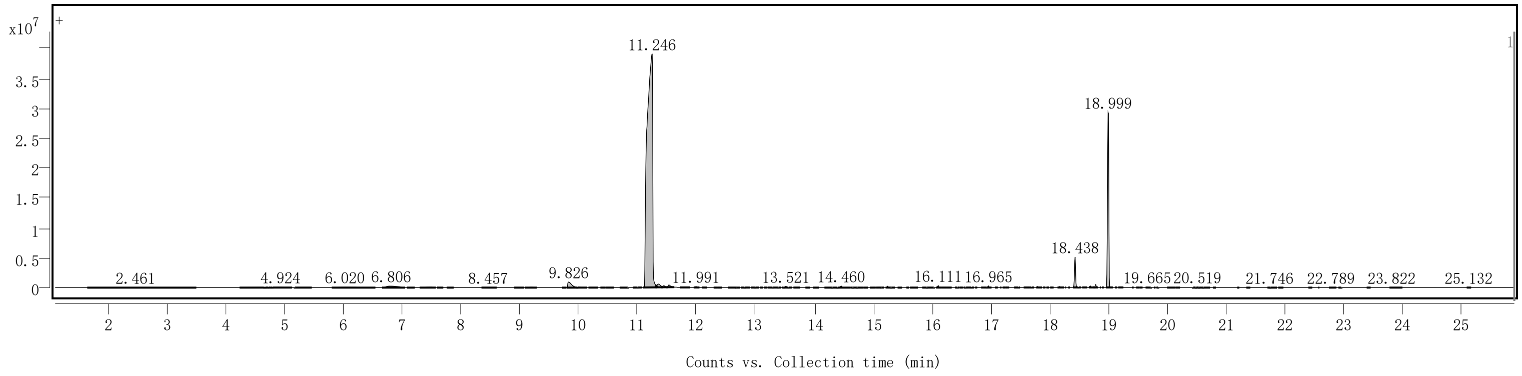

Chromatogram Peaks

Peak	Start	RT	End	Height	Area	Area %	SNR
1	1.638	2.461	3.493	20807	1148345	0.46	
2	4.227	4.620	4.647	45386	557695	0.22	
3	4.647	4.667	4.673	47090	66791	0.03	
4	4.673	4.715	4.720	56365	133397	0.05	
5	4.720	4.751	4.762	59923	133482	0.05	
6	4.762	4.783	4.788	62517	93110	0.04	
7	4.788	4.924	5.124	77402	889753	0.35	
8	5.165	5.328	5.456	16684	145193	0.06	
9	5.795	6.020	6.539	82777	1742135	0.69	
10	6.652	6.806	7.043	250058	3442823	1.37	
11	7.073	7.152	7.207	19321	99793	0.04	
12	7.290	7.477	7.567	91563	704487	0.28	
13	7.588	7.661	7.693	30967	98863	0.04	
14	7.750	7.776	7.870	10499	29847	0.01	
15	8.342	8.457	8.601	40974	343447	0.14	
16	8.899	9.008	9.071	23082	126393	0.05	
17	9.084	9.186	9.285	75130	442912	0.18	
18	9.714	9.763	9.789	2319	6546	0.00	
19	9.799	9.826	9.972	939477	4403353	1.75	
20	9.972	9.993	10.142	97271	354907	0.14	
21	10.161	10.219	10.325	9352	58108	0.02	
22	10.365	10.413	10.449	7434	22886	0.01	
23	10.449	10.533	10.594	26238	124527	0.05	
24	10.692	10.743	10.832	21427	95488	0.04	
25	10.915	10.953	11.000	88515	218628	0.09	
26	11.000	11.037	11.068	30334	57956	0.02	
27	11.086	11.246	11.314	39049498	251904444	100.00	
28	11.320	11.351	11.414	548017	2153372	0.85	
29	11.414	11.445	11.498	256111	789201	0.31	
30	11.498	11.529	11.622	372861	1104553	0.44	
31	11.722	11.760	11.833	19087	67499	0.03	
32	11.833	11.870	11.891	5868	13282	0.01	
33	11.950	11.991	12.050	147632	313147	0.12	
34	12.088	12.116	12.183	22024	59859	0.02	
35	12.291	12.316	12.352	6309	13250	0.01	
36	12.352	12.399	12.436	8389	18312	0.01	
37	12.537	12.578	12.667	22448	89132	0.04	
38	12.667	12.698	12.741	11873	24785	0.01	
39	12.757	12.793	12.819	56594	106327	0.04	
40	12.819	12.861	12.908	69346	186270	0.07	
41	12.924	12.955	12.997	52346	119929	0.05	
42	12.997	13.023	13.061	35873	74619	0.03	
43	13.079	13.107	13.132	22454	35909	0.01	
44	13.150	13.181	13.207	29347	48667	0.02	
45	13.213	13.243	13.296	20783	62088	0.02	
46	13.296	13.369	13.390	18774	58559	0.02	
47	13.390	13.422	13.442	22849	32115	0.01	
48	13.453	13.474	13.485	18789	22002	0.01	
49	13.485	13.521	13.563	216610	377124	0.15	
50	13.563	13.600	13.622	14745	30948	0.01	
51	13.647	13.668	13.763	43403	79510	0.03	
52	13.852	13.878	13.931	12660	28094	0.01	

Chromatogram Peaks

Peak	Start	RT	End	Height	Area	Area %	SNR
53	13.984	14.025	14.087	26443	65929	0.03	
54	14.175	14.224	14.255	9148	18549	0.01	
55	14.255	14.292	14.308	11482	19638	0.01	
56	14.308	14.344	14.397	14510	49999	0.02	
57	14.397	14.460	14.538	259508	615285	0.24	
58	14.538	14.580	14.617	10830	30098	0.01	
59	14.617	14.638	14.659	8087	15082	0.01	
60	14.659	14.690	14.732	23393	56972	0.02	
61	14.732	14.758	14.842	54809	101194	0.04	
62	14.842	14.879	14.913	5997	14444	0.01	
63	14.948	15.015	15.047	13942	49334	0.02	
64	15.056	15.083	15.133	26221	58152	0.02	
65	15.136	15.157	15.188	7360	12905	0.01	
66	15.215	15.251	15.309	228569	353584	0.14	
67	15.309	15.356	15.374	7769	12739	0.01	
68	15.419	15.471	15.498	8270	17734	0.01	
69	15.569	15.592	15.613	4234	5073	0.00	
70	15.630	15.676	15.764	64131	156823	0.06	
71	15.840	15.896	15.917	26213	48874	0.02	
72	15.917	15.985	16.037	43913	121560	0.05	
73	16.074	16.111	16.158	322386	454335	0.18	
74	16.159	16.189	16.242	20341	41221	0.02	
75	16.242	16.273	16.339	15273	39862	0.02	
76	16.395	16.415	16.436	17481	22012	0.01	
77	16.436	16.452	16.472	12919	14305	0.01	
78	16.473	16.504	16.528	9631	19419	0.01	
79	16.541	16.556	16.572	7166	7845	0.00	
80	16.572	16.604	16.619	6255	10186	0.00	
81	16.619	16.656	16.677	92137	130499	0.05	
82	16.677	16.698	16.723	23238	39866	0.02	
83	16.741	16.750	16.777	6984	7588	0.00	
84	16.842	16.855	16.871	9796	10893	0.00	
85	16.871	16.923	16.939	17336	42931	0.02	
86	16.939	16.965	17.018	265233	389664	0.15	
87	17.074	17.091	17.123	11763	18342	0.01	
88	17.167	17.175	17.187	11134	7514	0.00	
89	17.205	17.227	17.246	8802	11584	0.00	
90	17.251	17.269	17.280	9349	10395	0.00	
91	17.280	17.296	17.319	10356	13472	0.01	
92	17.400	17.458	17.474	21881	56518	0.02	
93	17.474	17.484	17.501	21617	19595	0.01	
94	17.563	17.579	17.657	61100	103821	0.04	
95	17.657	17.678	17.694	17958	20753	0.01	
96	17.694	17.715	17.740	21450	31215	0.01	
97	17.779	17.799	17.876	161220	263460	0.10	
98	17.894	17.898	17.935	5416	7032	0.00	
99	17.951	17.972	17.995	12747	17273	0.01	
100	18.015	18.029	18.045	11261	10864	0.00	
101	18.045	18.061	18.073	9668	9656	0.00	
102	18.140	18.176	18.249	165547	224257	0.09	
103	18.272	18.276	18.286	4046	803	0.00	
104	18.323	18.333	18.349	13481	12491	0.00	
105	18.402	18.438	18.496	5081210	6624423	2.63	
106	18.496	18.512	18.543	29672	52611	0.02	
107	18.559	18.580	18.645	82917	124711	0.05	
108	18.671	18.695	18.732	258002	350426	0.14	
109	18.732	18.753	18.769	124097	149306	0.06	
110	18.769	18.790	18.827	486630	598529	0.24	
111	18.858	18.868	18.879	5148	3775	0.00	
112	18.894	18.910	18.926	7362	7565	0.00	
113	18.962	18.999	19.079	29296626	44605038	17.71	
114	19.115	19.130	19.156	20336	29119	0.01	
115	19.173	19.198	19.261	72121	131229	0.05	
116	19.408	19.419	19.434	7758	6211	0.00	
117	19.476	19.497	19.534	8781	14605	0.01	
118	19.534	19.555	19.586	14289	20185	0.01	
119	19.637	19.665	19.728	89838	125874	0.05	
120	19.778	19.791	19.807	6095	6516	0.00	
121	19.822	19.838	19.854	4629	4270	0.00	
122	20.002	20.053	20.084	14825	26483	0.01	
123	20.084	20.116	20.147	33337	49433	0.02	
124	20.147	20.194	20.223	31359	47378	0.02	
125	20.441	20.467	20.493	112186	131196	0.05	
126	20.493	20.519	20.546	117869	151155	0.06	
127	20.546	20.567	20.619	55981	118529	0.05	
128	20.619	20.635	20.734	16883	43270	0.02	
129	20.782	20.802	20.833	8677	12014	0.00	
130	21.196	21.217	21.232	5939	5833	0.00	
131	21.352	21.400	21.419	11913	16268	0.01	
132	21.710	21.746	21.757	32486	49750	0.02	
133	21.757	21.778	21.872	24772	67206	0.03	
134	21.893	21.924	21.982	6835	16339	0.01	
135	22.407	22.428	22.472	12381	21229	0.01	
136	22.574	22.580	22.593	2991	1960	0.00	
137	22.756	22.789	22.883	20485	59305	0.02	
138	22.916	22.941	22.967	4103	6251	0.00	
139	23.397	23.439	23.465	5279	9911	0.00	

Analysis Report

Chromatogram Peaks

Peak	Start	RT	End	Height	Area	Area %	SNR
140	23.785	23.822	24.005	12486	49890	0.02	
141	25.098	25.132	25.174	5507	12563	0.00	
