# Supplementary material for: A Comparative Study Based on HS-SPME-GC-MS of Volatile Compounds in Large Yellow Croaker (Pseudosciaena crocea) During Varied Cold Storage Conditions
Source: Foods. 2025 Jun 11;14(12):2063. doi: 10.3390/foods14122063 (PMC12192311; doi:10.3390/foods14122063)
Supplement: Supplementary file 1 [file foods-14-02063-s001.zip › foods-3503473-supplementary/补充文件/P0 _Analysis-structure.template.pdf]

Analysis Report

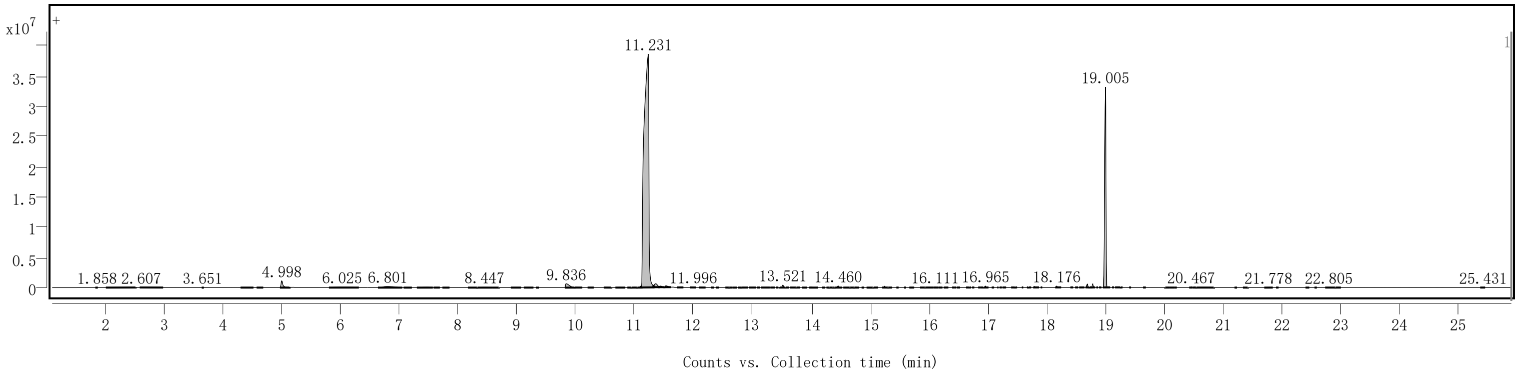

Chromatogram Peaks

Peak	Strat	RT	End	Height	Area	Area %	SNR
1	1.826	1.858	1.868	2908	4228	0.00	
2	2.006	2.351	2.513	15840	292816	0.14	
3	2.581	2.607	2.980	17311	226008	0.10	
4	3.635	3.651	3.672	2879	4241	0.00	
5	4.295	4.400	4.516	8902	57010	0.03	
6	4.568	4.610	4.682	6898	30278	0.01	
7	4.967	4.998	5.129	1126290	2793137	1.29	
8	5.800	6.025	6.308	111041	2102122	0.97	
9	6.635	6.801	7.047	193355	2594682	1.20	
10	7.068	7.158	7.210	16789	79923	0.04	
11	7.295	7.456	7.566	73571	610698	0.28	
12	7.577	7.656	7.686	18107	61928	0.03	
13	7.729	7.760	7.850	8322	27972	0.01	
14	8.165	8.274	8.321	10511	69923	0.03	
15	8.321	8.447	8.589	64302	558449	0.26	
16	8.589	8.646	8.688	8116	24024	0.01	
17	8.892	8.998	9.070	31182	184631	0.09	
18	9.114	9.181	9.277	44095	248100	0.11	
19	9.323	9.333	9.374	3284	5538	0.00	
20	9.811	9.836	9.978	637405	3029659	1.40	
21	9.978	10.004	10.108	72300	248160	0.11	
22	10.198	10.245	10.302	7593	25092	0.01	
23	10.472	10.539	10.602	41632	175527	0.08	
24	10.671	10.748	10.839	58585	253610	0.12	
25	10.874	10.911	10.942	30103	63030	0.03	
26	10.942	11.000	11.042	64729	201966	0.09	
27	11.042	11.100	11.115	228777	426601	0.20	
28	11.115	11.231	11.320	38529084	216564069	100.00	
29	11.320	11.356	11.414	568563	2169171	1.00	
30	11.414	11.440	11.503	184615	607454	0.28	
31	11.503	11.529	11.617	235419	746223	0.34	
32	11.721	11.771	11.828	20578	75313	0.03	
33	11.942	11.996	12.045	64792	178165	0.08	
34	12.091	12.132	12.205	29756	84393	0.04	
35	12.299	12.316	12.351	4118	7535	0.00	
36	12.378	12.400	12.431	5316	10610	0.00	
37	12.541	12.573	12.604	23821	54855	0.03	
38	12.604	12.625	12.662	17366	36414	0.02	
39	12.662	12.698	12.740	16942	31076	0.01	
40	12.755	12.793	12.809	51297	87761	0.04	
41	12.809	12.850	12.924	61516	198586	0.09	
42	12.942	13.023	13.068	42266	130934	0.06	
43	13.077	13.107	13.133	25079	39154	0.02	
44	13.147	13.175	13.212	33175	61917	0.03	
45	13.212	13.244	13.296	29471	80127	0.04	
46	13.307	13.348	13.381	54632	105607	0.05	
47	13.399	13.422	13.443	21374	29167	0.01	
48	13.464	13.521	13.563	385523	733419	0.34	
49	13.563	13.600	13.628	22361	53337	0.02	
50	13.645	13.668	13.747	60799	136743	0.06	
51	13.747	13.773	13.813	13455	30726	0.01	
52	13.846	13.883	13.932	14071	33871	0.02	

Chromatogram Peaks

Peak	Start	RT	End	Height	Area	Area %	SNR
53	13.968	13.993	14.114	116176	316489	0.15	
54	14.187	14.213	14.241	5673	10859	0.01	
55	14.261	14.287	14.308	23665	37279	0.02	
56	14.308	14.339	14.371	14209	42677	0.02	
57	14.371	14.386	14.402	12247	17506	0.01	
58	14.402	14.460	14.530	267073	580765	0.27	
59	14.548	14.580	14.659	7185	25932	0.01	
60	14.659	14.685	14.732	27371	62664	0.03	
61	14.732	14.759	14.818	105320	178982	0.08	
62	14.858	14.884	14.904	4294	6118	0.00	
63	14.943	15.015	15.052	32677	93374	0.04	
64	15.063	15.084	15.136	22171	50027	0.02	
65	15.210	15.251	15.309	211909	334441	0.15	
66	15.309	15.356	15.377	17614	31959	0.01	
67	15.450	15.477	15.488	8241	10223	0.00	
68	15.579	15.597	15.611	3804	4591	0.00	
69	15.671	15.718	15.755	14498	40924	0.02	
70	15.851	15.896	15.938	26252	53060	0.02	
71	15.938	15.985	16.043	54551	128103	0.06	
72	16.043	16.059	16.085	5936	9156	0.00	
73	16.085	16.111	16.155	102907	156224	0.07	
74	16.164	16.190	16.235	17630	30982	0.01	
75	16.254	16.279	16.335	12025	27632	0.01	
76	16.398	16.415	16.435	18485	22285	0.01	
77	16.436	16.452	16.467	10798	8577	0.00	
78	16.475	16.499	16.529	9399	20706	0.01	
79	16.632	16.656	16.688	91060	130564	0.06	
80	16.688	16.703	16.723	23205	30516	0.01	
81	16.738	16.751	16.777	13754	17210	0.01	
82	16.843	16.855	16.871	12891	11566	0.01	
83	16.874	16.918	16.934	14952	27963	0.01	
84	16.934	16.965	17.017	222673	335556	0.15	
85	17.070	17.091	17.112	20140	27722	0.01	
86	17.167	17.180	17.187	7452	4758	0.00	
87	17.206	17.222	17.242	12324	14137	0.01	
88	17.255	17.264	17.317	10721	22966	0.01	
89	17.402	17.453	17.469	24593	65807	0.03	
90	17.469	17.484	17.503	40830	44501	0.02	
91	17.582	17.594	17.610	10285	8816	0.00	
92	17.668	17.684	17.694	17604	18295	0.01	
93	17.694	17.715	17.744	30065	47009	0.02	
94	17.779	17.799	17.873	148001	274957	0.13	
95	17.904	17.919	17.933	8822	7006	0.00	
96	18.152	18.176	18.254	211539	270572	0.12	
97	18.410	18.438	18.459	103784	130630	0.06	
98	18.489	18.517	18.543	27855	42271	0.02	
99	18.560	18.580	18.649	77081	122679	0.06	
100	18.666	18.695	18.732	562307	730925	0.34	
101	18.732	18.753	18.769	117437	150007	0.07	
102	18.769	18.790	18.833	568804	678852	0.31	
103	18.852	18.863	18.879	6680	6687	0.00	
104	18.895	18.915	18.938	10636	12614	0.01	
105	18.960	19.005	19.086	33086147	51293668	23.69	
106	19.116	19.136	19.153	20645	23741	0.01	
107	19.167	19.209	19.267	39512	88840	0.04	
108	19.267	19.277	19.297	18849	17224	0.01	
109	19.408	19.419	19.440	14677	11254	0.01	
110	19.644	19.665	19.691	72292	87210	0.04	
111	20.022	20.058	20.090	12819	23488	0.01	
112	20.090	20.116	20.174	18953	33494	0.02	
113	20.174	20.195	20.218	31512	40387	0.02	
114	20.430	20.467	20.488	136377	154433	0.07	
115	20.488	20.520	20.551	120516	161937	0.07	
116	20.551	20.577	20.755	27827	160458	0.07	
117	20.755	20.803	20.850	10013	24051	0.01	
118	21.201	21.217	21.242	5812	6025	0.00	
119	21.343	21.358	21.379	5295	6080	0.00	
120	21.379	21.400	21.426	11885	15683	0.01	
121	21.708	21.778	21.851	29651	103121	0.05	
122	21.906	21.924	21.951	5122	6886	0.00	
123	22.409	22.449	22.473	5638	9211	0.00	
124	22.562	22.580	22.600	5834	7671	0.00	
125	22.742	22.805	22.915	13316	68539	0.03	
126	22.915	22.947	23.010	7325	26015	0.01	
127	25.379	25.431	25.463	4853	14049	0.01	
