# Supplementary material for: A Comparative Study Based on HS-SPME-GC-MS of Volatile Compounds in Large Yellow Croaker (Pseudosciaena crocea) During Varied Cold Storage Conditions
Source: Foods. 2025 Jun 11;14(12):2063. doi: 10.3390/foods14122063 (PMC12192311; doi:10.3390/foods14122063)
Supplement: Supplementary file 1 [file foods-14-02063-s001.zip › foods-3503473-supplementary/补充文件/P12 _Analysis-structure.template.pdf]

Analysis Report

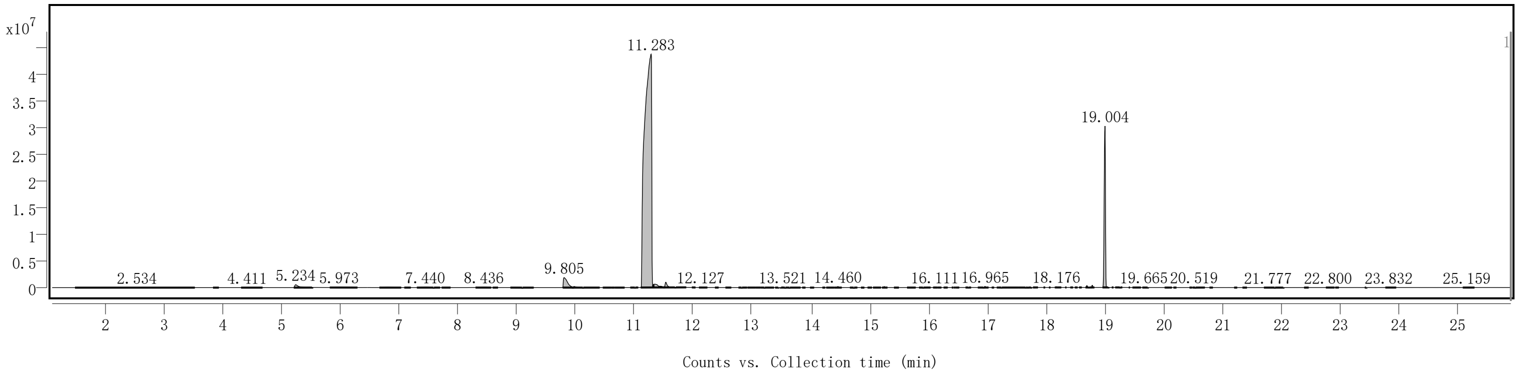

Chromatogram Peaks

Peak	Start	RT	End	Height	Area	Area %	SNR
1	1.481	2.534	3.519	34682	2045579	0.57	
2	3.829	3.865	3.928	4893	12349	0.00	
3	4.306	4.411	4.672	6699	74119	0.02	
4	5.203	5.234	5.517	549717	2829017	0.79	
5	5.815	5.973	6.287	78968	1452424	0.40	
6	6.659	6.801	7.030	75179	991750	0.28	
7	7.083	7.152	7.194	4695	16949	0.00	
8	7.297	7.440	7.582	90858	844846	0.24	
9	7.582	7.661	7.692	21925	84163	0.02	
10	7.718	7.797	7.870	6836	32116	0.01	
11	8.295	8.436	8.562	75530	631813	0.18	
12	8.586	8.630	8.678	7959	26368	0.01	
13	8.888	8.971	9.092	42322	262465	0.07	
14	9.092	9.176	9.285	24603	161542	0.04	
15	9.778	9.805	9.951	1871337	9156489	2.55	
16	9.951	9.972	10.124	212309	793517	0.22	
17	10.124	10.187	10.407	25259	215026	0.06	
18	10.460	10.533	10.601	17034	79320	0.02	
19	10.602	10.633	10.664	6761	16350	0.00	
20	10.664	10.753	10.832	22853	99768	0.03	
21	10.928	10.937	11.010	33334	83752	0.02	
22	11.010	11.036	11.063	19695	35010	0.01	
23	11.105	11.283	11.320	43803261	359276592	100.00	
24	11.320	11.346	11.503	635285	3897789	1.08	
25	11.503	11.529	11.692	975672	2724489	0.76	
26	11.697	11.739	11.880	40595	166234	0.05	
27	11.975	12.001	12.038	28901	58359	0.02	
28	12.096	12.127	12.239	72892	195681	0.05	
29	12.368	12.389	12.431	8397	15665	0.00	
30	12.546	12.583	12.646	13477	39389	0.01	
31	12.768	12.798	12.824	18843	36348	0.01	
32	12.824	12.855	12.913	17401	50444	0.01	
33	12.918	13.018	13.065	31863	133900	0.04	
34	13.065	13.107	13.149	22068	50096	0.01	
35	13.149	13.175	13.212	23536	50130	0.01	
36	13.212	13.259	13.374	26766	109000	0.03	
37	13.392	13.422	13.448	25173	42540	0.01	
38	13.490	13.521	13.563	126249	240328	0.07	
39	13.563	13.605	13.637	18360	45227	0.01	
40	13.637	13.668	13.826	57510	165156	0.05	
41	13.853	13.883	13.909	8812	15044	0.00	
42	13.986	14.019	14.056	8333	15686	0.00	
43	14.196	14.224	14.245	5930	10077	0.00	
44	14.261	14.339	14.397	15266	60669	0.02	
45	14.397	14.460	14.526	200128	437574	0.12	
46	14.664	14.685	14.732	11432	24506	0.01	
47	14.732	14.758	14.790	58322	89127	0.02	
48	14.853	14.884	14.910	5474	10062	0.00	
49	14.965	15.020	15.040	13758	35821	0.01	
50	15.057	15.083	15.193	39808	100807	0.03	
51	15.209	15.251	15.304	196075	306455	0.09	
52	15.414	15.471	15.497	8416	19078	0.01	

Chromatogram Peaks

Peak	Start	RT	End	Height	Area	Area %	SNR
53	15.637	15.691	15.784	20734	81357	0.02	
54	15.839	15.891	15.912	16031	33881	0.01	
55	15.912	15.980	16.037	58359	171142	0.05	
56	16.079	16.111	16.158	117272	174240	0.05	
57	16.158	16.184	16.221	13214	24181	0.01	
58	16.259	16.273	16.328	12251	22322	0.01	
59	16.397	16.410	16.429	14106	15541	0.00	
60	16.436	16.452	16.466	9474	8479	0.00	
61	16.470	16.488	16.524	8930	12817	0.00	
62	16.631	16.656	16.682	85721	116140	0.03	
63	16.682	16.703	16.726	28716	45492	0.01	
64	16.840	16.923	16.939	28423	77615	0.02	
65	16.939	16.965	17.023	205734	322170	0.09	
66	17.076	17.091	17.117	11837	15806	0.00	
67	17.163	17.175	17.191	7330	7451	0.00	
68	17.191	17.217	17.243	17551	33344	0.01	
69	17.243	17.264	17.337	19435	67337	0.02	
70	17.337	17.484	17.531	29328	175503	0.05	
71	17.531	17.584	17.657	45928	159423	0.04	
72	17.657	17.678	17.694	36817	60984	0.02	
73	17.694	17.715	17.752	41928	94352	0.03	
74	17.779	17.799	17.863	132145	255935	0.07	
75	17.958	17.972	17.986	8837	8145	0.00	
76	18.050	18.061	18.077	8686	6420	0.00	
77	18.150	18.176	18.260	141921	199011	0.06	
78	18.327	18.339	18.349	10511	7325	0.00	
79	18.413	18.438	18.461	79587	100918	0.03	
80	18.491	18.517	18.538	22349	34050	0.01	
81	18.563	18.580	18.609	55182	62402	0.02	
82	18.671	18.695	18.732	356365	464646	0.13	
83	18.732	18.753	18.769	119804	143312	0.04	
84	18.769	18.789	18.824	404890	517888	0.14	
85	18.962	19.004	19.062	30274295	45825028	12.75	
86	19.119	19.135	19.148	15404	13767	0.00	
87	19.177	19.204	19.261	73239	146621	0.04	
88	19.261	19.277	19.298	26352	31562	0.01	
89	19.408	19.419	19.429	10814	6880	0.00	
90	19.471	19.555	19.615	15778	62114	0.02	
91	19.639	19.665	19.733	69461	100810	0.03	
92	20.019	20.053	20.095	12776	22851	0.01	
93	20.095	20.121	20.142	18123	26213	0.01	
94	20.168	20.194	20.220	36793	48022	0.01	
95	20.441	20.467	20.488	99211	113297	0.03	
96	20.493	20.519	20.540	110064	131835	0.04	
97	20.540	20.577	20.703	52907	195546	0.05	
98	20.781	20.802	20.839	10232	16434	0.00	
99	21.201	21.217	21.253	6280	8127	0.00	
100	21.342	21.400	21.419	16195	28577	0.01	
101	21.708	21.777	21.893	40069	186940	0.05	
102	21.893	21.919	22.040	9706	35423	0.01	
103	22.391	22.448	22.469	8271	16910	0.00	
104	22.761	22.800	22.910	24575	88811	0.02	
105	22.920	22.941	22.983	6909	14403	0.00	
106	23.423	23.434	23.450	5113	5402	0.00	
107	23.773	23.832	23.958	14008	60637	0.02	
108	25.091	25.159	25.290	7573	46497	0.01	
