# Supplementary material for: A Comparative Study Based on HS-SPME-GC-MS of Volatile Compounds in Large Yellow Croaker (Pseudosciaena crocea) During Varied Cold Storage Conditions
Source: Foods. 2025 Jun 11;14(12):2063. doi: 10.3390/foods14122063 (PMC12192311; doi:10.3390/foods14122063)
Supplement: Supplementary file 1 [file foods-14-02063-s001.zip › foods-3503473-supplementary/补充文件/P6 _Analysis-structure.template.pdf]

Analysis Report

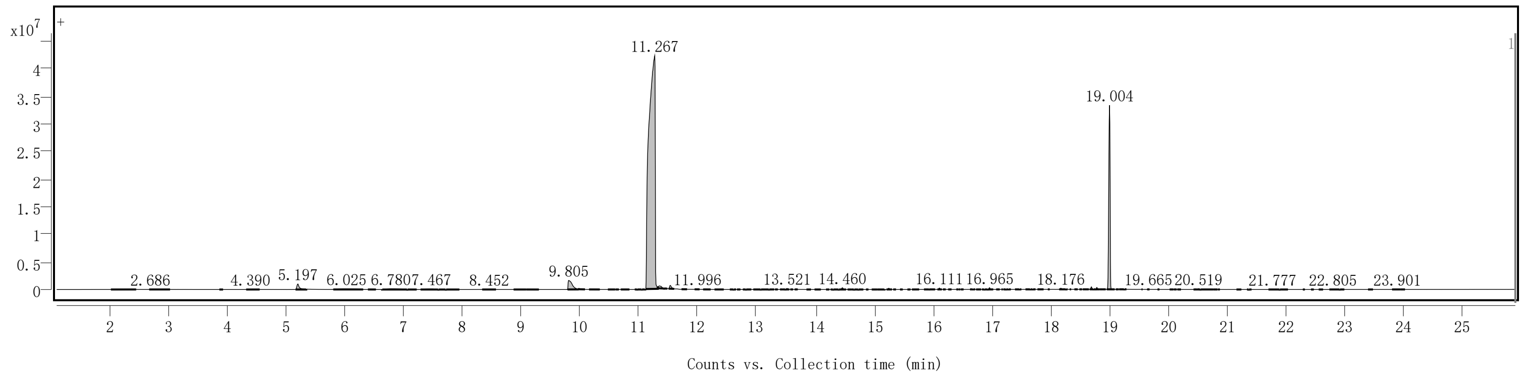

Chromatogram Peaks

Peak	Start	RT	End	Height	Area	Area %	SNR
1	2.015	2.382	2.445	4917	90203	0.03	
2	2.666	2.686	3.021	11703	101581	0.03	
3	3.860	3.897	3.923	3567	5277	0.00	
4	4.317	4.390	4.546	8727	50299	0.02	
5	5.156	5.197	5.338	1000351	3218547	1.05	
6	5.798	6.025	6.307	115915	2140042	0.70	
7	6.388	6.486	6.523	11027	46360	0.02	
8	6.628	6.780	7.058	140485	1909559	0.62	
9	7.058	7.152	7.218	12793	65321	0.02	
10	7.283	7.467	7.587	95317	920840	0.30	
11	7.587	7.666	7.713	25054	110460	0.04	
12	7.713	7.781	7.944	11407	75317	0.02	
13	8.332	8.452	8.566	28602	226766	0.07	
14	8.866	8.987	9.081	27449	153792	0.05	
15	9.081	9.176	9.296	34046	219980	0.07	
16	9.784	9.805	9.957	1629370	7984655	2.60	
17	9.957	9.978	10.080	133177	384688	0.13	
18	10.149	10.193	10.333	15802	102494	0.03	
19	10.470	10.544	10.596	19936	86709	0.03	
20	10.601	10.617	10.657	6309	10288	0.00	
21	10.689	10.753	10.837	25715	114058	0.04	
22	10.928	10.942	10.968	36615	66087	0.02	
23	10.968	10.995	11.047	42729	121513	0.04	
24	11.047	11.089	11.105	27024	63933	0.02	
25	11.115	11.267	11.320	42192005	306569649	100.00	
26	11.320	11.346	11.424	444427	1780884	0.58	
27	11.424	11.445	11.481	93048	191305	0.06	
28	11.503	11.529	11.599	599422	1360566	0.44	
29	11.723	11.744	11.818	20335	70635	0.02	
30	11.946	11.996	12.030	48611	108068	0.04	
31	12.090	12.143	12.219	17529	58901	0.02	
32	12.284	12.310	12.352	4157	8603	0.00	
33	12.352	12.389	12.441	15464	38213	0.01	
34	12.545	12.572	12.609	18899	39660	0.01	
35	12.609	12.625	12.651	8708	14865	0.00	
36	12.667	12.704	12.725	10218	17454	0.01	
37	12.767	12.793	12.819	26781	48736	0.02	
38	12.819	12.850	12.910	23382	69673	0.02	
39	12.943	13.018	13.070	45067	154793	0.05	
40	13.070	13.112	13.144	26989	58636	0.02	
41	13.144	13.175	13.212	18205	38711	0.01	
42	13.212	13.243	13.301	26112	80289	0.03	
43	13.314	13.348	13.361	6992	8421	0.00	
44	13.399	13.422	13.448	21127	34149	0.01	
45	13.448	13.469	13.490	19243	28311	0.01	
46	13.490	13.521	13.556	175811	315206	0.10	
47	13.574	13.600	13.621	13539	22652	0.01	
48	13.647	13.668	13.700	40952	65499	0.02	
49	13.849	13.878	13.925	8089	20549	0.01	
50	13.987	14.030	14.093	20323	60098	0.02	
51	14.182	14.218	14.237	4363	7838	0.00	
52	14.260	14.287	14.302	11377	15950	0.01	

Chromatogram Peaks

Peak	Start	RT	End	Height	Area	Area %	SNR
53	14.302	14.350	14.397	10419	41471	0.01	
54	14.397	14.460	14.523	315184	668611	0.22	
55	14.549	14.570	14.612	7520	15323	0.00	
56	14.612	14.643	14.659	6949	10606	0.00	
57	14.659	14.685	14.732	14414	33076	0.01	
58	14.732	14.758	14.816	57543	93026	0.03	
59	14.861	14.884	14.903	3967	4463	0.00	
60	14.959	15.015	15.052	17298	58772	0.02	
61	15.052	15.083	15.178	31824	108485	0.04	
62	15.210	15.251	15.304	221221	331896	0.11	
63	15.325	15.356	15.372	6176	10457	0.00	
64	15.440	15.471	15.487	7206	9466	0.00	
65	15.566	15.592	15.618	8296	13918	0.00	
66	15.644	15.718	15.765	15124	54476	0.02	
67	15.848	15.891	15.938	16081	37701	0.01	
68	15.938	15.980	16.037	48641	103021	0.03	
69	16.076	16.111	16.152	254651	351915	0.11	
70	16.170	16.189	16.218	11569	14952	0.00	
71	16.260	16.305	16.317	5043	10888	0.00	
72	16.398	16.410	16.432	16616	17215	0.01	
73	16.438	16.452	16.465	10554	9000	0.00	
74	16.474	16.488	16.522	7549	12836	0.00	
75	16.631	16.656	16.682	89345	119605	0.04	
76	16.682	16.703	16.719	17863	27012	0.01	
77	16.735	16.750	16.820	15441	40684	0.01	
78	16.834	16.855	16.876	21490	29094	0.01	
79	16.876	16.918	16.934	20642	50506	0.02	
80	16.934	16.965	17.026	307676	464259	0.15	
81	17.076	17.096	17.136	13541	21043	0.01	
82	17.165	17.175	17.189	7031	4815	0.00	
83	17.198	17.217	17.243	15210	23901	0.01	
84	17.243	17.269	17.285	16432	25007	0.01	
85	17.285	17.296	17.326	13412	20063	0.01	
86	17.396	17.453	17.474	28181	73331	0.02	
87	17.474	17.484	17.504	16783	18079	0.01	
88	17.575	17.594	17.657	19487	52189	0.02	
89	17.657	17.678	17.699	22911	34842	0.01	
90	17.699	17.715	17.746	21137	33204	0.01	
91	17.778	17.799	17.883	132537	261358	0.09	
92	17.955	17.972	17.987	13085	11097	0.00	
93	18.147	18.176	18.265	210653	307109	0.10	
94	18.265	18.281	18.291	8695	8009	0.00	
95	18.327	18.339	18.351	11381	8144	0.00	
96	18.407	18.438	18.463	59303	79181	0.03	
97	18.491	18.517	18.538	21138	32537	0.01	
98	18.548	18.580	18.653	82728	139479	0.05	
99	18.666	18.695	18.732	447177	591716	0.19	
100	18.732	18.753	18.769	127876	162711	0.05	
101	18.769	18.790	18.837	340345	442035	0.14	
102	18.837	18.868	18.884	9824	14688	0.00	
103	18.884	18.905	18.940	13068	26062	0.01	
104	18.957	19.004	19.093	33298430	53039284	17.30	
105	19.112	19.136	19.162	27760	39253	0.01	
106	19.168	19.204	19.256	55209	129379	0.04	
107	19.256	19.282	19.293	15602	19294	0.01	
108	19.549	19.555	19.566	5418	2250	0.00	
109	19.645	19.665	19.685	58064	64500	0.02	
110	19.820	19.843	19.854	4066	5042	0.00	
111	20.027	20.053	20.092	12581	17949	0.01	
112	20.097	20.121	20.147	25413	32367	0.01	
113	20.158	20.194	20.220	30991	43022	0.01	
114	20.433	20.467	20.493	101576	119011	0.04	
115	20.493	20.519	20.551	149781	195733	0.06	
116	20.551	20.577	20.771	47286	228775	0.07	
117	20.771	20.808	20.845	11799	24024	0.01	
118	20.851	20.876	20.882	3291	3660	0.00	
119	21.164	21.217	21.247	6509	10431	0.00	
120	21.342	21.363	21.374	9028	10518	0.00	
121	21.374	21.395	21.421	13239	18885	0.01	
122	21.708	21.777	21.898	41727	178435	0.06	
123	21.898	21.924	22.040	12544	37810	0.01	
124	22.293	22.312	22.328	3628	3519	0.00	
125	22.433	22.448	22.479	6878	9654	0.00	
126	22.564	22.585	22.637	5569	11309	0.00	
127	22.744	22.805	22.920	18072	104040	0.03	
128	22.920	22.936	22.999	8450	24364	0.01	
129	23.403	23.434	23.486	5927	11623	0.00	
130	23.811	23.901	23.953	7566	36361	0.01	
131	23.953	23.974	24.032	6188	16251	0.01	
